# Integrated plasma proteomics identifies HLA-G and osteopontin as circulating biomarkers of resistance to PD-1 inhibitor plus chemotherapy in choriocarcinoma

**DOI:** 10.3389/fimmu.2026.1817862

**Published:** 2026-08-04

**Authors:** Weidi Wang, Tianyi Chen, Haoyu Wang, Jiayuan Zhao, Chen Yang, Yuxiao Wu, Yujia Kong, Dan Wang, Yuan Li, Junjun Yang, Yang Xiang

**Affiliations:** National Clinical Research Center for Women’s Health and Obstetric and Gynecologic Diseases, Department of Obstetrics and Gynecology, Peking Union Medical College Hospital, Chinese Academy of Medical Sciences & Peking Union Medical College, Beijing, China

**Keywords:** choriocarcinoma, gestational trophoblastic neoplasia, HLA-G, osteopontin, PD-1 inhibitor, plasma proteomics

## Abstract

**Introduction:**

Although programmed cell death protein 1 (PD-1) inhibitors combined with chemotherapy have improved outcomes in high-risk choriocarcinoma, primary resistance remains a clinically important challenge without reliable predictive biomarkers.

**Methods:**

We performed data-independent acquisition mass spectrometry (DIA-MS) on pretreatment plasma from a nested discovery subset of 30 patients, followed by enzyme-linked immunosorbent assay (ELISA) assessment of shortlisted candidates in the full cohort of 60 patients. An exploratory two-marker logistic model was developed and internally assessed in the same cohort. Exploratory tissue immunophenotyping and analyses of public tissue-transcriptomic and immunotherapy datasets were used to provide biological context.

**Results:**

DIA-MS identified a resistance-associated plasma proteomic signature enriched in inflammatory and immunomodulatory mediators among patients with progressive disease. In the full-cohort ELISA assessment, elevated baseline plasma HLA-G and osteopontin (OPN) were associated with primary resistance. The exploratory two-marker model showed an apparent area under the receiver operating characteristic curve of 0.908. In the limited tissue subset, higher HLA-G expression tended to be associated with lower CD8-positive T-cell density, whereas higher OPN expression was associated with increased CD163-positive macrophage density. Public-dataset analyses provided supportive context for associations of HLA-G and SPP1 transcript expression with immune-related programs and unfavorable outcomes.

**Discussion:**

Elevated circulating HLA-G and OPN are associated with resistance to PD-1 inhibitor plus chemotherapy in choriocarcinoma and may serve as candidate minimally invasive biomarkers for pretreatment risk stratification. Independent validation and functional investigation are required before clinical application.

## 1 Introduction

Gestational trophoblastic neoplasia (GTN) encompasses a unique group of pregnancy-related malignancies, including invasive mole, choriocarcinoma, placental site trophoblastic tumor (PSTT), and epithelioid trophoblastic tumor (ETT) (1). While choriocarcinoma is generally considered highly curable due to its high sensitivity to chemotherapy, the management of high-risk patients remains a significant clinical challenge (2). For decades, multi-agent regimens such as EMA/CO have served as the cornerstone of treatment, achieving high remission rates in appropriately treated patients (3). However, approximately 20–30% of high-risk patients develop resistance to first-line multi-agent chemotherapy (4), and multi-drug resistant disease remains a leading cause of cancer-related mortality in this population (5). Furthermore, the cumulative toxicity of intensive chemotherapy highlights the need for more effective and less toxic treatment strategies (2).

The inherent immunogenicity of trophoblastic tumors, characterized by their semi-allogeneic genomic origin and high expression of programmed death-ligand 1 (PD-L1), has provided a strong biological rationale for immune checkpoint blockade (ICB) (6). Clinical studies have shown activity of PD-1 inhibitor–based combinations in patients with chemorefractory GTN, with retrospective data suggesting higher response rates for PD-1 blockade plus chemotherapy than for immunotherapy alone (7–10). Despite these advances, clinical outcomes are heterogeneous: a subset of patients exhibits primary resistance despite combination therapy. Crucially, unlike in other solid tumors, PD-L1 expression is ubiquitous in choriocarcinoma (>90%) and lacks discriminative power as a predictive biomarker for treatment failure (6, 11). Consequently, clinicians currently lack reliable biological tools to identify non-responders early. Because diagnosis often relies on serology and imaging and pretreatment tissue is frequently unavailable (1, 2), tissue-based biomarkers (e.g., tumor mutational burden or microsatellite instability) are difficult to implement (12), underscoring the need for blood-based biomarkers that may capture treatment-relevant biological states.

The failure of PD-1 blockade in PD-L1-positive tumors suggests the presence of alternative immune evasion pathways that prevent T-cell infiltration or function. Trophoblasts possess unique immune-modulatory properties physiologically designed to protect the fetus from maternal rejection, a process involving the expression of the non-classical MHC class I molecule HLA-G (13). Emerging evidence indicates that HLA-G can inhibit NK- and CD8+ T-cell effector functions (14, 15). Concurrently, the tumor microenvironment (TME) of resistant tumors is often remodeled by myeloid cells. Osteopontin (OPN/SPP1) has been implicated in macrophage recruitment and M2-like polarization (16, 17) and has been associated with immunosuppressive tumor microenvironments (18, 19). Previous studies have implicated HLA-G and SPP1 in treatment resistance or immunosuppressive tissue states (20, 21), but whether circulating HLA-G and OPN are associated with primary resistance to PD-1 inhibitor plus chemotherapy in choriocarcinoma remains unclear.

In this study, we used a nested plasma-proteomic discovery and full-cohort ELISA assessment to identify pretreatment circulating proteins associated with primary resistance to PD-1 inhibitor plus chemotherapy in choriocarcinoma. We further explored their associations with tissue immune-cell features and immune-related transcriptional programs in public datasets.

## 2 Materials and methods

### 2.1 Patient enrollment and study design

This retrospective study was approved by the Ethics Committee of Peking Union Medical College Hospital (No. K4907). All participants provided written informed consent. We enrolled a total of 60 patients with choriocarcinoma who received PD-1 inhibitor therapy (camrelizumab or toripalimab) combined with chemotherapy between January 2022 and June 2025. The inclusion criteria were: (1) histologically or clinically diagnosed choriocarcinoma according to FIGO diagnostic criteria (based on serum β-hCG dynamics and imaging findings); (2) classified as high-risk (FIGO prognostic score ≥7) at the time of initiating PD-1–based chemo-immunotherapy, assessed at the most recent clinical evaluation prior to treatment (typically within 1–2 weeks); (3) received at least two cycles of PD-1 inhibitor plus chemotherapy and (4) evaluable disease according to RECIST v1.1 or elevated serum β-hCG levels. The exclusion criteria were clinically significant comorbidities, autoimmune disease, or previous treatment with an immune checkpoint inhibitor. Patients were stratified into two groups based on their response to chemo-immunotherapy: Complete Response (CR; serological and/or radiological remission) and Progressive Disease (PD; earliest confirmed radiologic progression and/or sustained rising β-hCG), as defined in **Supplementary Methods S1**, with the timing of PD determination clearly specified.

**Figure 1:**
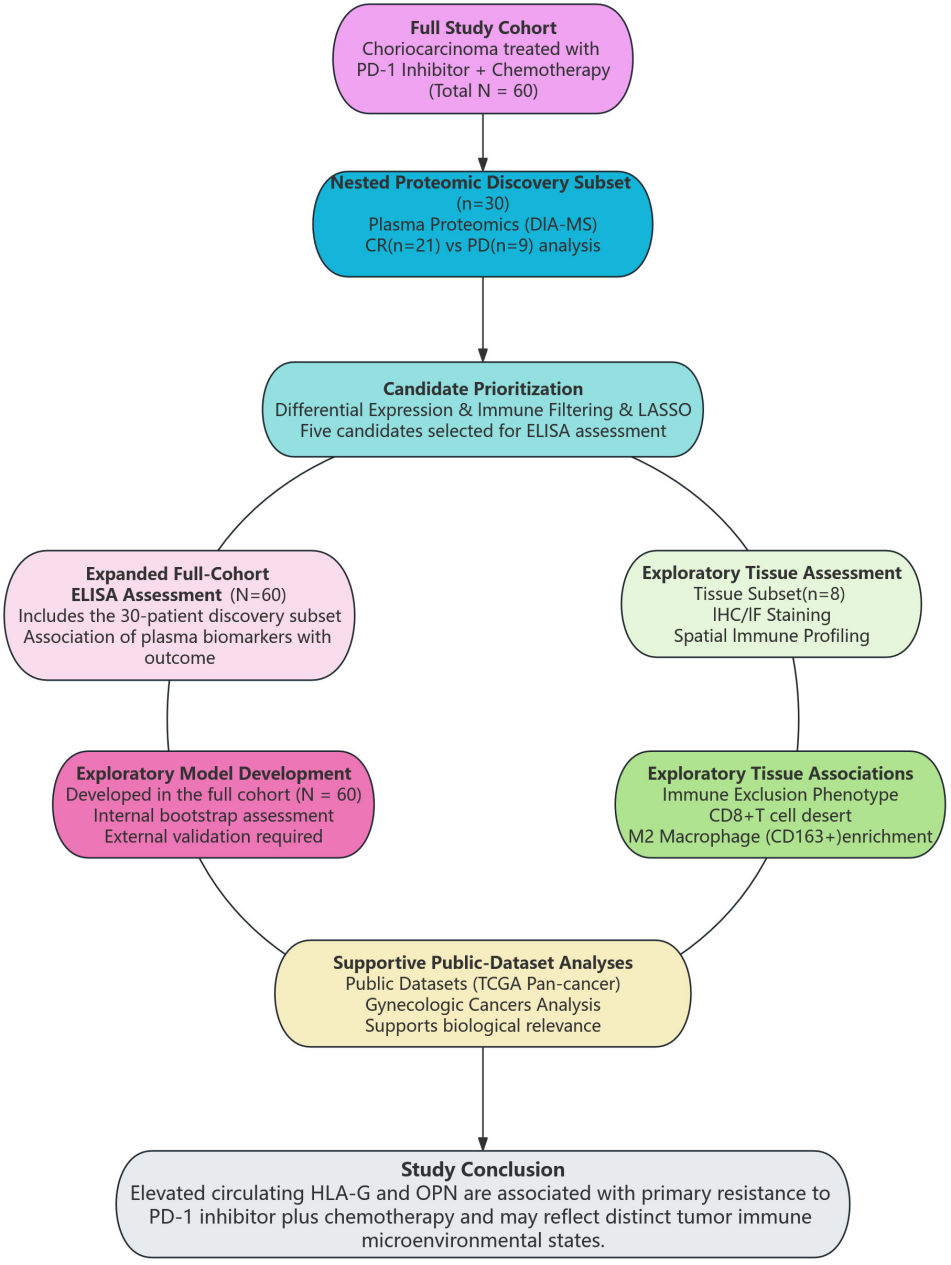
Study design and analytical workflow. The study included a full cohort of 60 patients with choriocarcinoma treated with a PD-1 inhibitor plus chemotherapy (CR, n = 47; PD, n = 13). A nested subset of 30 patients from this full cohort (CR, n = 21; PD, n = 9) was selected for pretreatment plasma proteomic discovery using DIA-MS. Candidate proteins identified in the discovery subset were subsequently quantified by ELISA in the full cohort of 60 patients. Thus, the full-cohort ELISA assessment included the 30 patients used for proteomic discovery and did not constitute an independent validation cohort. An exploratory two-marker model was developed and internally assessed in the same full cohort. Available surgical tissues from an exploratory subset of eight patients were analyzed by IHC and duplex IF, and public transcriptomic and immunotherapy datasets were used to provide supportive biological context. CR, complete response; PD, progressive disease; PD-1, programmed cell death protein 1; DIA–MS, data-independent acquisition mass spectrometry; LASSO, least absolute shrinkage and selection operator; HLA-G, human leukocyte antigen G; OPN, osteopontin; SPP1, secreted phosphoprotein 1; ELISA, enzyme-linked immunosorbent assay; IHC, immunohistochemistry; IF, immunofluorescence.

The study included a full cohort of 60 patients, comprising 47 patients who achieved complete response and 13 patients who developed progressive disease. Within this cohort, a nested subset of 30 patients (21 CR and 9 PD) was selected for pretreatment plasma proteomic discovery using DIA-MS. Candidate proteins identified in this discovery subset were subsequently quantified by ELISA in the full cohort of 60 patients. Thus, the full-cohort ELISA analysis included the 30 patients used for proteomic discovery and represented an expanded internal assessment rather than an independent validation cohort. In addition, archival surgical tumor tissues were available from an exploratory subset of eight patients (4 CR and 4 PD) for tissue immunophenotyping (**Figure 1**).

### 2.2 Plasma proteomics (DIA-MS) and data analysis

Pretreatment peripheral blood samples were collected in EDTA-coated tubes. Plasma was separated by centrifugation at 2000×g for 10 minutes and stored at -80 °C. Proteins were enriched using magnetic TiO_2_ beads [1:4, w/w protein:beads; modified from Montoya et al., 2011 (22)] with sequential washes. Enrichment efficiency and protein extraction recovery were verified using spiked peptide internal standards, achieving >90% recovery, before digestion on-bead with trypsin (37 °C, 1, 500 rpm, ≥3 h). Peptides were desalted (spin columns), vacuum-dried, reconstituted in 8 μL mobile phase A (0.1% FA in water), and spiked with iRT peptides (Biognosys; 1:20, v/v) prior to injection. DIA-MS was performed on a timsTOF HT (Bruker) coupled to an UltiMate 3000 nanoLC (Thermo Fisher Scientific) with a C18 column (15 cm × 100 μm, 1.9 μm) using 0.1% FA in water (A) and 0.1% FA in acetonitrile (B). Data were processed with Spectronaut Pulsar v18.7 against the uniprot-Homo sapiens-9606-irt-2024.2.1.fasta database (Trypsin/P; ≤ 2 missed cleavages; fixed carbamidomethyl-C; variable oxidation-M and protein N-terminal acetylation). Q-value cutoffs were 0.01 at precursor and protein levels, with MS2-based quantification.

Differential abundance analysis was performed using limma on log2-transformed protein intensities. Differentially expressed proteins (DEPs) were defined by |log2 fold change| ≥1 and Benjamini–Hochberg FDR <0.05. To characterize biological alterations, Gene Ontology (GO) and Kyoto Encyclopedia of Genes and Genomes (KEGG) over-representation analyses were first performed separately for the full sets of upregulated and downregulated DEPs using all proteins retained for differential analysis. Gene Set Enrichment Analysis (GSEA) was performed on the complete protein list ranked by the limma moderated t-statistic using Human Phenotype Ontology (HPO) gene sets. Multiple-testing correction was applied to all enrichment analyses.

After completion of these global analyses, DEPs were intersected with the MSigDB Gene Ontology “immune response” gene set (GOBP_IMMUNE_RESPONSE; GO:0006955) to define an immune-focused subset for hypothesis-driven candidate prioritization. Protein-protein interaction (PPI) networks were built in STRING (v11.5; confidence score >0.4) and visualized with Cytoscape (v3.9.1), utilizing the MCODE plugin to identify dense molecular complexes.

### 2.3 Enzyme-linked immunosorbent assay

Plasma concentrations of candidate biomarkers were quantified using commercial ELISA kits according to the manufacturers’ instructions. The specific kits used were: HLA-G (Elabscience, E-EL-H1663), CXCL12/SDF-1 (Abcam, ab100637), osteopontin (OPN/SPP1) (Bioswamp, HM10043), PD-L1 (Bioswamp, HM10627), and IL-6R (Bioswamp, HM10449). Absorbance was measured at 450 nm using a microplate reader (Multiskan SkyHigh, Thermo Scientific). All samples were analyzed in duplicate using a randomized plate layout to minimize plate position effects, and the mean concentration was used for downstream analyses. Plasma aliquots were thawed once on ice and clarified by brief centrifugation prior to assay. Concentrations were calculated from standard curves (4-parameter logistic fitting), and samples exceeding the upper limit of quantification were diluted and re-assayed. Laboratory operators were blinded to clinical outcomes during the assays. Assay performance characteristics are provided in **Supplementary Methods S2**.

### 2.4 Immunohistochemistry and scoring

FFPE tissue sections (4 µm) were deparaffinized and rehydrated. Antigen retrieval was performed in EDTA buffer (pH 9.0). Endogenous peroxidase activity was quenched with 3% H_2_O_2_ for 10 min at room temperature, followed by protein blocking with 1% BSA in PBS containing 0.3% Triton X-100 (30 min, room temperature). Sections were incubated with primary antibodies against HLA-G (mouse monoclonal, clone 1E5A10, Proteintech, 66447-1-Ig; dilution 1:200) or OPN (rabbit recombinant monoclonal, clone 240206A2, Proteintech, 83341-1-RR; dilution 1:400) overnight at 4 °C, followed by incubation with polymer-based HRP detection system (Dako) according to the manufacturer’s instructions. Staining was visualized using DAB substrate (3–5 min), counterstained with hematoxylin, dehydrated, and mounted.

IHC expression was quantified using the H-score method. Whole-slide images were analyzed in QuPath (version 0.6.0) using color deconvolution (H-DAB) and predefined intensity thresholds to classify tumor cells as 0 (negative), 1+ (weak), 2+ (moderate), or 3+ (strong). The H-score was calculated as H-score = Σ(i×Pi), where i denotes staining intensity (0–3) and Pi is the percentage of tumor cells at each intensity (0–100%), yielding a range of 0–300. Tumor regions of interest were annotated and quality-controlled by two investigators blinded to clinical outcomes, and discrepant annotations/threshold settings were resolved by consensus. Intensity thresholds were calibrated using negative-control slides and low-signal regions and were applied consistently across slides.

### 2.5 Immunofluorescence staining and quantitative analysis

Duplex immunofluorescence staining was performed on FFPE tissue sections (4 μm). Slides were deparaffinized, rehydrated, and subjected to heat-induced antigen retrieval in EDTA buffer (pH 9.0). After blocking with 1% BSA in PBS containing 0.3% Triton X-100 (30 min, room temperature), sections were incubated with primary antibodies overnight at 4 °C. To maintain consistency with IHC, the same primary antibodies were used for HLA-G and OPN/SPP1: HLA-G (clone 1E5A10) and OPN/SPP1 (clone 240206A2).

For immune cell phenotyping, duplex staining was performed using CD8 (rabbit monoclonal, clone D8A8Y, Cell Signaling Technology, 85336S; dilution 1:200) and CD163 (mouse monoclonal, clone 10D6, Novus Biologicals, NB110-59935; dilution 1:100) antibodies validated for FFPE immunofluorescence. Two duplex panels were performed: HLA-G (red) with CD8 (green) and OPN/SPP1 (red) with CD163 (green). Species-specific fluorophore-conjugated secondary antibodies were applied for 1 h at room temperature (Alexa Fluor 594, 1:500 for the red channel; Alexa Fluor 488, 1:500 for the green channel). Nuclei were counterstained with DAPI (1 µg/mL, 5 min), and slides were mounted with an antifade medium. Images were acquired using a whole-slide fluorescence scanner (ZEISS Axioscan 7, Carl Zeiss; 20× objective) and analyzed using digital pathology software (QuPath, version 0.6.0). Single-stain controls were used to set acquisition parameters and to minimize spectral bleed-through between channels.

Tumor regions of interest (ROIs) were delineated by referencing matched H&E sections and corresponding DAPI/tumor morphology on immunofluorescence images. Viable tumor areas were defined as regions containing morphologically identifiable choriocarcinoma cells with preserved nuclear architecture. Areas of necrosis, hemorrhage, extensive fibrosis, tissue folds, edge artifacts, and non-tumor stromal or inflammatory regions were excluded. ROI annotation was performed on whole-slide images using QuPath by two investigators blinded to clinical outcomes, and discrepant annotations were resolved by consensus.

Within tumor ROIs, HLA-G and OPN signals were quantified as background-corrected mean fluorescence intensity (MFI), where background was defined as the median signal from adjacent non-tumor stromal areas within the same section and subtracted from tumor ROI measurements. For immune-cell quantification, intratumoral compartments were defined as viable tumor-cell regions within the annotated tumor boundary, whereas peritumoral compartments were defined as a 200 μm stromal band extending outward from the tumor invasive margin. CD8+ T cells and CD163+ macrophages were quantified separately in intratumoral and peritumoral compartments as cell density (cells/mm²), followed by manual quality control.

### 2.6 Public database analyses (TCGA and pan-immunotherapy)

Pan-cancer gene expression profiles were obtained from the TCGA PanCancer Atlas harmonized RNA-seq matrix (EBPlusPlusAdjustPANCAN_IlluminaHiSeq_RNASeqV2.geneExp.tsv) (23), and clinical survival annotations were retrieved from the UCSC Xena “phenotype—Curated clinical data” resource. Expression and clinical data were matched by sample identifiers. Additional details are provided in **Supplementary Methods S3**.

To characterize immune associations, correlations between target gene expression and published immune signature scores (24) were computed within specific gynecologic cancer types (BRCA, CESC, OV, UCEC). In addition, Spearman correlations between HLA-G or SPP1 expression and selected immunomodulatory genes (25) were assessed within the same four cancer types. For visualization, a representative subset of biologically relevant suppressive and co-stimulatory genes was displayed. For the pan-immunotherapy overall survival analysis, multiple pretreatment ICB cohorts were integrated: within each cohort, gene expression was Z-score standardized, outliers were removed, and cohorts were pooled for Kaplan–Meier estimation with log-rank testing.

These analyses were performed to provide supportive biological context and were not intended as external validation of the circulating biomarker associations.

### 2.7 Statistical analysis

All analyses were performed in R (v4.2.0) and GraphPad Prism (v9.0). Continuous variables are summarized as median (IQR) and compared using the Mann–Whitney U test; categorical variables are summarized as n (%) and compared using the χ² test or Fisher’s exact test, as appropriate. Correlations between continuous variables were assessed using Spearman’s rank correlation. All tests were two-sided, and P < 0.05 was considered statistically significant.

In the nested proteomic discovery subset, protein intensities were analyzed on the log2 scale, and differential abundance testing was performed using limma with empirical Bayes moderation. Multiple testing was controlled using the Benjamini–Hochberg method. Among immune-related upregulated DEPs, LASSO logistic regression (glmnet) with 3-fold cross-validation was used to select candidate biomarkers, with features retained at the λ1se penalty. In the full cohort, which included the proteomic discovery subset, shortlisted candidates were quantified by ELISA and evaluated using group comparisons and logistic regression. HLA-G and OPN, which showed significant associations in the full-cohort ELISA assessment, were included in the exploratory two-marker model. A multivariable logistic regression model was fitted with primary resistance (PD) as the outcome and pretreatment plasma HLA-G (ng/mL) and OPN (μg/L) as predictors (original measurement units). Given the limited number of PD events (n = 13), the two-marker model was considered exploratory. The two-marker biomarker score was defined as the model linear predictor: LP=β0 + β1×HLA-G + β2×OPN, and the predicted probability of PD was obtained by the inverse-logit transformation: P(PD) = 1/[1 + exp(-LP)]. For comparability of effect sizes, we additionally reported standardized effects by refitting the model using Z-score–standardized biomarkers z=(x−μ)/σ (μ and σ computed in the full cohort), yielding odds ratios per 1-SD increase (**Supplementary Table 6**). A nomogram was constructed to visually illustrate the relationship between biomarker values and model-estimated probability. Discrimination was assessed using ROC curves and AUC. Model performance was internally assessed using 1, 000 bootstrap resamples to obtain 95% CIs, optimism-corrected performance and bootstrap calibration. Potential net benefit within the study cohort was explored using decision curve analysis (DCA), and continuous (category-free) net reclassification improvement (NRI) was calculated to quantify the incremental value of the combined model over single-marker models. A cohort-derived exploratory cutoff was selected using the Youden index.

Progression-free survival (PFS) was estimated using the Kaplan–Meier method and compared using the log-rank test, with patients stratified by the cohort-derived exploratory cutoff. To explore potential clinical confounding, Spearman correlations were calculated between HLA-G or OPN and selected continuous clinical variables. Given the limited number of PD events, separate parsimonious logistic models were fitted for each biomarker with one clinical covariate at a time, including FIGO score, pretreatment β-hCG, tumor diameter, metastatic burden and sites, prior chemotherapy failure, chemotherapy regimen, PD-1 inhibitor type, and disease status. These analyses were exploratory and are reported in Supplementary_Confounding_Analyses.

## 3 Results

### 3.1 Cohort characteristics

Among the 60 included patients, 47 (78.3%) achieved CR and 13 (21.7%) developed PD. No statistically significant differences were detected in the measured baseline variables; however, the small sample size and several numerical imbalances precluded exclusion of residual confounding (all P>0.05; **Table 1**). The potential influence of selected clinical factors was explored in subsequent sensitivity analyses (Supplementary_Confounding_Analyses). A nested subset of 30 patients (21 CR and 9 PD) from this full cohort was selected for proteomic discovery. Clinical characteristics in this proteomic discovery subset also showed no statistically significant differences between response groups (all P > 0.05; **Supplementary Table 1**).

**Table 1:** Baseline characteristics of the full cohort of patients with choriocarcinoma treated with PD-1 inhibitor plus chemotherapy, stratified by treatment outcome (CR vs PD).

Characteristic^1^	CR (n=47)	PD (n=13)	P value^2^
Disease status			0.478
Primary	37 (78.7)	9 (69.2)	
Recurrent	10 (21.3)	4 (30.8)	
Age, years	33 [30, 38]	30 [29, 37]	0.364
FIGO stage			0.292
I	6 (12.8)	1 (7.7)	
II	2 (4.3)	1 (7.7)	
III	22 (46.8)	3 (23.1)	
IV	17 (36.2)	8 (61.5)	
FIGO prognostic score	12 [9.5, 14]	15 [13, 17]	0.068
Antecedent pregnancy			0.738
Molar pregnancy	16 (34.0)	3 (23.1)	
Term delivery	17 (36.2)	6 (46.2)	
Abortion/miscarriage	14 (29.8)	4 (30.8)	
Interval from antecedent pregnancy to treatment, months	26 [12, 48]	40 [15, 60]	0.355
Pretreatment serum β-hCG, IU/L	53, 281 [8, 528, 278, 664]	113, 576 [2, 125, 225, 000]	0.554
Maximum tumor diameter, cm	4.7 [2.5, 7.1]	6.0 [4.2, 6.5]	0.350
Pelvic metastasis			1.000
Yes	8 (17.0)	2 (15.4)	
No	39 (83.0)	11 (84.6)	
Lung metastasis			0.711
Yes	38 (80.9)	10 (76.9)	
No	9 (19.1)	3 (23.1)	
Brain metastasis			0.163
Yes	11 (23.4)	6 (46.2)	
No	36 (76.6)	7 (53.8)	
Liver metastasis			0.675
Yes	7 (14.9)	3 (23.1)	
No	40 (85.1)	10 (76.9)	
Other metastatic sites^3^			0.271
Yes	8 (17.0)	4 (30.8)	
No	39 (83.0)	9 (69.2)	
Number of metastatic lesions	1 [0, 4]	3 [1, 9]	0.104
History of chemotherapy failure			0.101
Yes	17 (36.2)	8 (61.5)	
No	30 (63.8)	5 (38.5)	
Chemotherapy regimen			0.242
EMA/CO	25 (53.2)	7 (53.8)	
FAEV	16 (34.0)	2 (15.4)	
Other^4^	6 (12.8)	4 (30.8)	
Immunotherapy agent			0.058
Camrelizumab	39 (83.0)	7 (53.8)	
Toripalimab	8 (17.0)	6 (46.2)	
Surgery performed			0.859
Yes	23 (48.9)	6 (46.2)	
No	24 (51.1)	7 (53.8)	

^1^ Data are presented as median [interquartile range] for continuous variables and n (%) for categorical variables. P values were two-sided.

^2^ Statistical tests by variable. 1. Continuous variables were compared using the Mann -Whitney U test: Age, FIGO prognostic score, Interval from antecedent pregnancy to treatment, Pretreatment serum hCG (IU/L), Maximum tumor diameter (cm), and Number of metastatic lesions. 2. Categorical variables were compared using Pearson’s chi-square test for History of chemotherapy failure and Surgery performed.3. Categorical variables were compared using Fisher’s exact test for Disease status, Lung metastasis, Pelvic metastasis, Brain metastasis, Liver metastasis, and Other metastatic sites, and Immunotherapy agent.4. For categorical variables with >2 categories, the Fisher -Freeman -Halton exact test was used, including Antecedent pregnancy, FIGO stage, and Chemotherapy regimen.

^3^ 12/60 patients had other metastatic sites; 3 patients had ≥2 other metastatic sites recorded. These included metastases to the kidney (n = 4), gastrointestinal tract (n = 3), spleen (n = 1), pancreas (n = 1), breast (n = 1), heart (n = 1), muscle (n = 1), spinal cord (n = 1), supraclavicular lymph node (n = 1), and sacrum (n = 1).

^4^ In Chemotherapy regimen, Other included EMA/EP (n=4), TP/TE (n=4), and KSM (n=2).

CR, complete response; PD, progressive disease; FIGO, International Federation of Gynecology and Obstetrics; hCG, human chorionic gonadotropin; IU/L, international units per liter; EMA/CO, etoposide + methotrexate + actinomycin D, alternating with cyclophosphamide + vincristine; FAEV, floxuridine (FUDR) + actinomycin D + etoposide + vincristine; EMA/EP, etoposide + methotrexate + actinomycin D, alternating with etoposide + cisplatin; TP/TE, paclitaxel + cisplatin, alternating with paclitaxel + etoposide. KSM, actinomycin D.

### 3.2 Plasma proteomics reveals an immune-resistance signature

**Figure 2:**
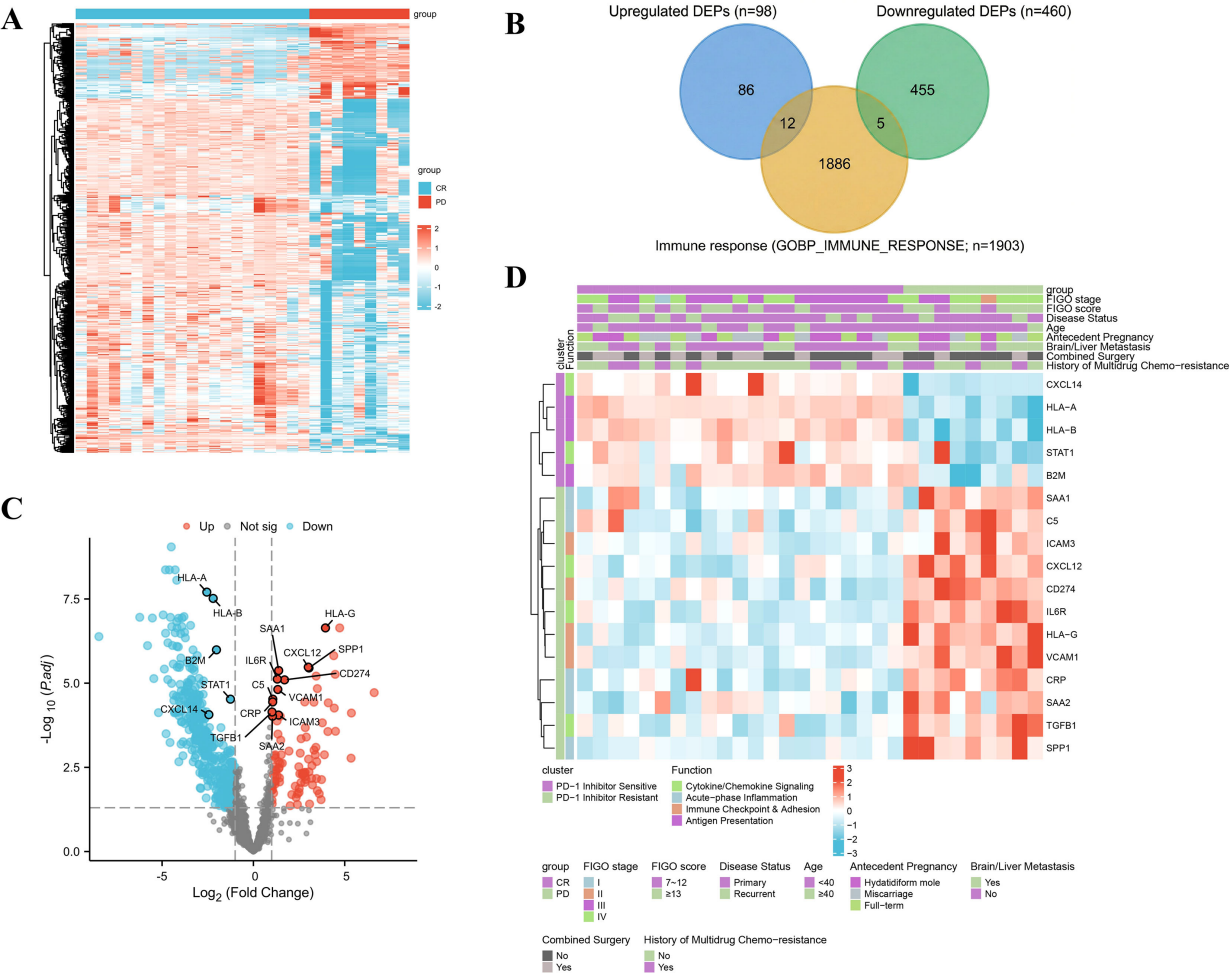
Plasma proteomics reveals an immune-resistance signature in choriocarcinoma treated with a PD-1 inhibitor plus chemotherapy. **(A)** Unsupervised hierarchical clustering heatmap of differentially expressed proteins in the nested proteomic discovery subset (n = 30; CR, n = 21; PD, n = 9) profiled by data-independent acquisition mass spectrometry. Protein abundances are scaled by row (z-score) and samples are annotated by response group. **(B)** Venn diagram showing overlap between proteins upregulated (n = 98) or downregulated (n = 460) in progressive disease versus complete response and an immune-response gene set (Gene Ontology Biological Process: immune response, GOBP_IMMUNE_RESPONSE), yielding 17 immune-related differentially expressed proteins (12 upregulated and 5 downregulated in progressive disease). The immune-response overlap shown in panel B was performed after the global enrichment analyses and was used only for hypothesis-driven candidate prioritization; it did not define the protein sets used for GO, KEGG, or GSEA. **(C)** Volcano plot summarizing differential abundance between progressive disease and complete response (x-axis, log2 fold change; y-axis, −log10 false discovery rate-adjusted P value). Selected immune-regulatory candidates are labeled, including human leukocyte antigen G and osteopontin/secreted phosphoprotein 1, together with additional inflammatory and adhesion-related mediators. **(D)** Focused heatmap of the 17 immune-related differentially expressed proteins across the discovery cohort with accompanying clinical annotations (including FIGO stage and score, disease status, age, antecedent pregnancy, metastatic sites, surgery, and history of multidrug chemo-resistance). Hierarchical clustering highlights a progressive disease–enriched immune-resistance pattern. CR, complete response; PD, progressive disease; PD-1, programmed cell death protein 1; DEP, differentially expressed protein; FDR, false discovery rate; GO, Gene Ontology; GOBP, Gene Ontology Biological Process; FIGO, International Federation of Gynecology and Obstetrics. Gene/protein symbols (full names): HLA-G, human leukocyte antigen G; SPP1/OPN, secreted phosphoprotein 1/osteopontin; CD274/PD-L1, programmed death-ligand 1; IL6R, interleukin-6 receptor; CXCL12/SDF-1, C-X-C motif chemokine ligand 12/stromal cell-derived factor 1; VCAM1, vascular cell adhesion molecule 1; ICAM3, intercellular adhesion molecule 3; TGFB1, transforming growth factor beta 1; CRP, C-reactive protein; SAA1/2, serum amyloid A1/2; B2M, beta-2-microglobulin; STAT1, signal transducer and activator of transcription 1; C5, complement component 5; HLA-A/B, human leukocyte antigen A/B.

We performed deep plasma proteomic profiling on pretreatment samples from the nested proteomic discovery subset. Hierarchical clustering of the identified DEPs showed response-associated abundance patterns across the discovery subset (**Figure 2A**). Using a predefined differential-expression threshold (|log2 Fold Change| ≥1 and adjusted P < 0.05), we identified 558 differentially expressed proteins (DEPs), including 98 upregulated and 460 downregulated proteins in PD relative to CR. To assess the robustness of candidate selection, we additionally performed sensitivity analyses using alternative differential-expression thresholds (|log2FC| ≥0.5, ≥1.5, and FDR <0.05 or <0.01). HLA-G and OPN remained significantly upregulated across all tested thresholds (**Supplementary Table 12**), supporting their prioritization for subsequent full-cohort assessment and exploratory model development. These full DEP sets were first subjected to global functional enrichment analyses, as described in Section 3.3. For subsequent hypothesis-driven candidate prioritization, we intersected DEPs with the MSigDB Gene Ontology “immune response” gene set (GOBP_IMMUNE_RESPONSE; GO:0006955; 1, 903 genes), identifying 17 immune-related DEPs (12 upregulated and 5 downregulated in PD) (**Figure 2B**; **Supplementary Table 2**). Among the proteins increased in resistant patients were key immunomodulatory and inflammatory mediators, including HLA-G, SPP1 (OPN), CD274 (PD-L1), IL6R, and CXCL12 (SDF-1), together with additional inflammation- and adhesion-related factors (e.g., SAA1, CRP, TGFB1, VCAM1 and ICAM3) (**Figure 2C**). Consistently, a focused heatmap of the immune-related DEPs showed coordinated upregulation in a PD-enriched cluster, with clinical variables shown as accompanying annotations to facilitate visual comparison across patient subgroups (**Figure 2D**).

Protein–protein interaction analysis identified an immune-related module enriched for upregulated proteins, in which HLA-G and SPP1 ranked among the most connected nodes. Complementary network analyses placed HLA-G and SPP1 within broader inflammatory and immune-regulatory programs (**Supplementary Figures 1A–C**).

### 3.3 Functional enrichment identifies resistance-associated immune and inflammatory pathways

**Figure 3:**
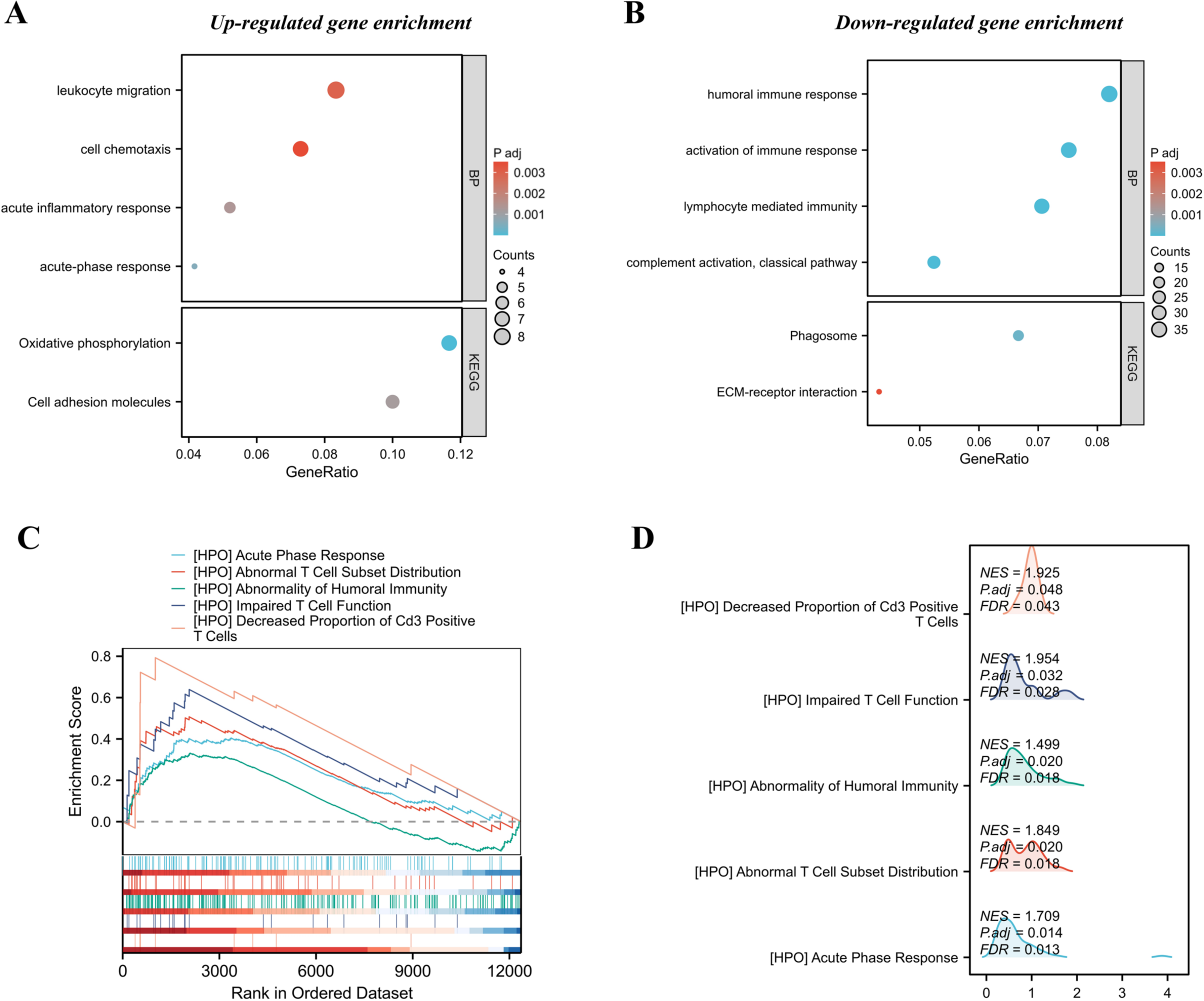
Functional enrichment of differentially expressed proteins highlights immune dysregulation associated with primary resistance. **(A)** Gene Ontology Biological Process (GO: BP) and Kyoto Encyclopedia of Genes and Genomes (KEGG) enrichment of proteins upregulated in progressive disease relative to complete response in the nested proteomic discovery subset. Dot size indicates the number of mapped proteins (Counts), dot color indicates the Benjamini–Hochberg adjusted P value, and the x axis shows GeneRatio. **(B)** GO: BP and KEGG enrichment of proteins downregulated in progressive disease relative to complete response, displayed as in **(A)**. **(C)** Gene set enrichment analysis (GSEA) enrichment plots for selected Human Phenotype Ontology (HPO) immune-related gene sets, showing enrichment across the ranked protein list. **(D)** Summary of GSEA results for the HPO terms shown in **(C)**, reporting normalized enrichment score (NES), adjusted P value and false discovery rate (FDR). GO: BP, Gene Ontology Biological Process; KEGG, Kyoto Encyclopedia of Genes and Genomes; GSEA, gene set enrichment analysis; HPO, Human Phenotype Ontology; NES, normalized enrichment score; FDR, false discovery rate.

To contextualize the proteomic alterations associated with resistance, we performed functional enrichment analyses on the full sets of 98 upregulated and 460 downregulated DEPs. GO and KEGG enrichment of proteins upregulated in the progressive disease (PD) group highlighted inflammatory programs, including terms related to the acute inflammatory response and leukocyte migration/chemotaxis, together with enrichment of oxidative phosphorylation–associated pathways (**Figure 3A**). In contrast, proteins downregulated in the PD group were enriched in immune-related categories, including humoral immune response, complement activation and lymphocyte-mediated immunity, with additional KEGG signals in phagosome and extracellular matrix–receptor interaction (**Figure 3B**), suggesting an association with attenuated immune-related programs in resistant disease.

To complement the over-representation analysis, we performed GSEA across the full ranked protein list. Among the significantly enriched HPO terms, several immune-related signatures were observed. Resistant patients showed significant enrichment of immune dysfunction–related signatures, including “Impaired T cell function” (NES = 1.95, FDR = 0.028) and “Abnormality of humoral immunity” (NES = 1.49, FDR = 0.018), and the HPO term “Reduced proportion of CD3-positive T cells” (**Figures 3C, D**). Collectively, these results support that baseline resistance to PD-1 inhibitor–based chemo-immunotherapy is associated with a circulating proteomic state characterized by inflammatory remodeling and alterations in adaptive immune-related programs.

Complete enrichment results are provided in Supplementary_GO_KEGG_UP, Supplementary_GO_KEGG_DOWN, and Supplementary_GSEA.

### 3.4 Full-cohort ELISA assessment supports associations of HLA-G and OPN with primary resistance

**Figure 4:**
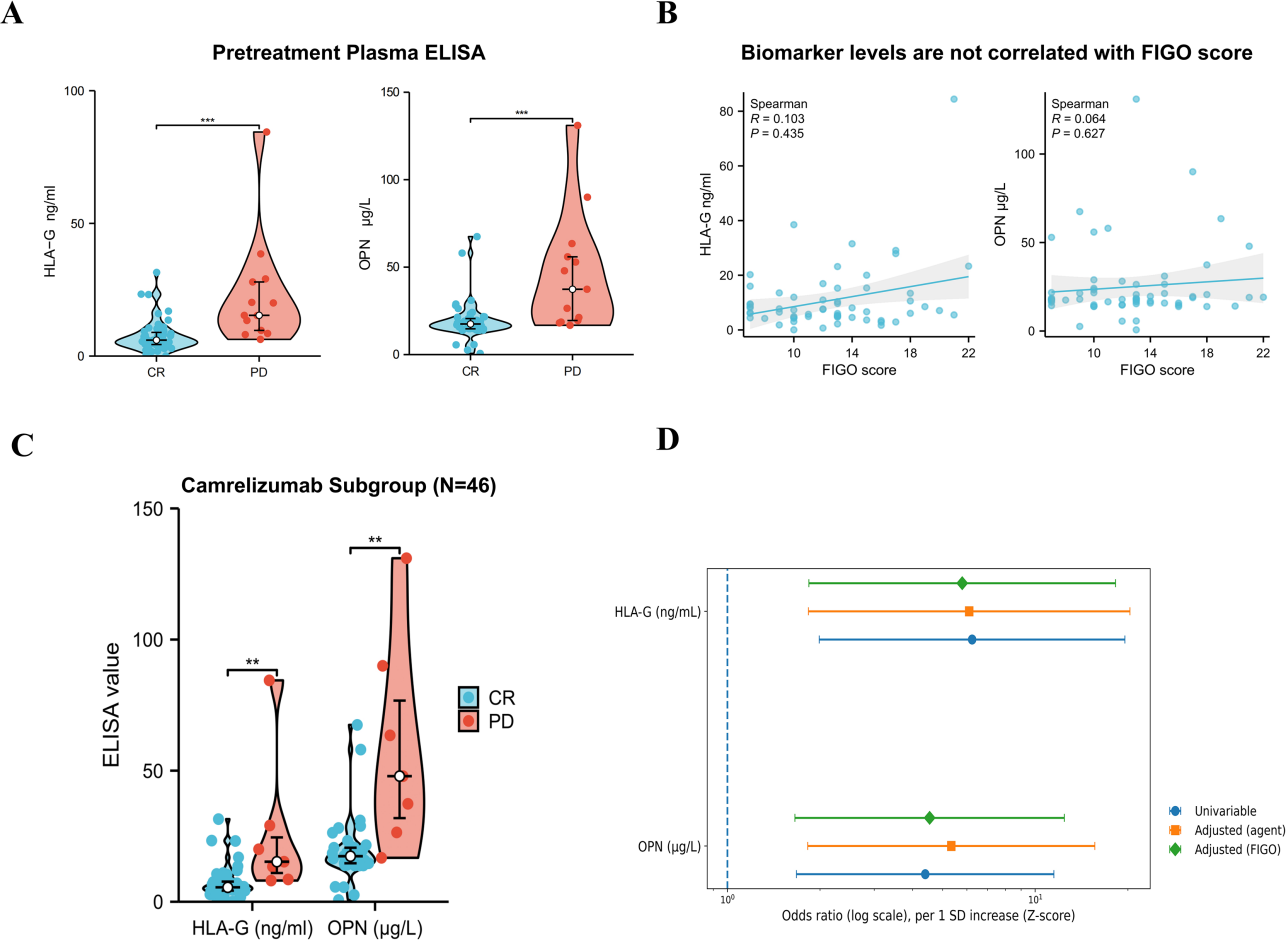
Pretreatment plasma HLA-G and OPN levels and exploratory analyses of selected clinical factors. **(A)** Pretreatment plasma concentrations of human leukocyte antigen G (HLA-G; ng/mL) and osteopontin (OPN; μg/L) measured by enzyme-linked immunosorbent assay (ELISA) in the full cohort (N = 60), stratified by treatment outcome (complete response (CR) versus progressive disease (PD)). Violin plots show the distribution with individual data points; center line indicates the median and error bars indicate the interquartile range. **(B)** Spearman rank correlations between pretreatment biomarker levels (HLA-G and OPN) and International Federation of Gynecology and Obstetrics (FIGO) prognostic score, with fitted regression line and 95% confidence interval. **(C)** Sensitivity analysis restricted to patients receiving camrelizumab (camrelizumab subgroup, N = 46), showing pretreatment HLA-G and OPN levels by outcome (CR versus PD). **(D)** Forest plot showing odds ratios (log scale) for primary resistance per 1 standard deviation increase in z-score–standardized HLA-G or OPN, estimated by logistic regression in univariable models and separate models adjusted individually for PD-1 inhibitor type or FIGO prognostic score. Two-sided P values were calculated using the Mann–Whitney U test for group comparisons (CR vs PD) in panels **(A)** and **(C)**, and Spearman’s rank correlation (two-sided) for associations in panel **(B)**. Significance is denoted as **P < 0.01 and ***P < 0.001. ELISA, enzyme-linked immunosorbent assay; HLA-G, human leukocyte antigen G; OPN, osteopontin; CR, complete response; PD, progressive disease; FIGO, International Federation of Gynecology and Obstetrics.

LASSO analysis of the immune-related upregulated DEPs prioritized five candidates for ELISA assessment: HLA-G, SPP1/OPN, CXCL12/SDF-1, IL6R, and CD274/PD-L1 (**Supplementary Figure 2**). In the full cohort, pretreatment plasma HLA-G and OPN levels were significantly higher in patients with PD than in those with CR (HLA-G: median 15.35 vs. 6.00 ng/mL; OPN: median 37.35 vs. 17.49 μg/L; both P < 0.001) (**Figure 4A**; **Supplementary Table 3**). In contrast, CXCL12, IL6R, and PD-L1 showed no significant between-group differences in the full cohort (all P > 0.05; **Supplementary Table 3**). To compare effect sizes on a unified scale and evaluate their association with resistance, we further standardized ELISA measurements to Z-scores and performed logistic regression analyses. Each 1 SD increase in HLA-G and OPN was associated with higher odds of resistance in univariable models, and both remained statistically associated with PD in the exploratory two-marker model (HLA-G: OR 7.07, 95% CI 1.74–28.77, P = 0.006; OPN: OR 4.41, 95% CI 1.43–13.57, P = 0.010) (**Supplementary Table 4**).

We next performed exploratory sensitivity analyses to assess the potential influence of selected clinical risk, disease-burden, and treatment-related factors. First, neither HLA-G nor OPN showed a strong correlation with FIGO prognostic score in this cohort (HLA-G: Spearman R = 0.103, P = 0.435; OPN: R = 0.064, P = 0.627) (**Figure 4B**). Second, in the camrelizumab-treated subgroup (n = 46), both HLA-G and OPN remained higher in patients with PD, showing directionally consistent associations within this subgroup (**Figure 4C**; **Supplementary Table 5**). Third, in separate limited covariate-adjusted logistic regression models, the associations of HLA-G and OPN with PD remained directionally consistent after adjustment for selected measures of disease burden, metastatic sites, prior chemotherapy failure, chemotherapy regimen, PD-1 inhibitor type, and disease status. Detailed results are provided in Supplementary_Confounding_Analyses.

### 3.5 Exploratory development and internal assessment of a two-marker model

**Figure 5:**
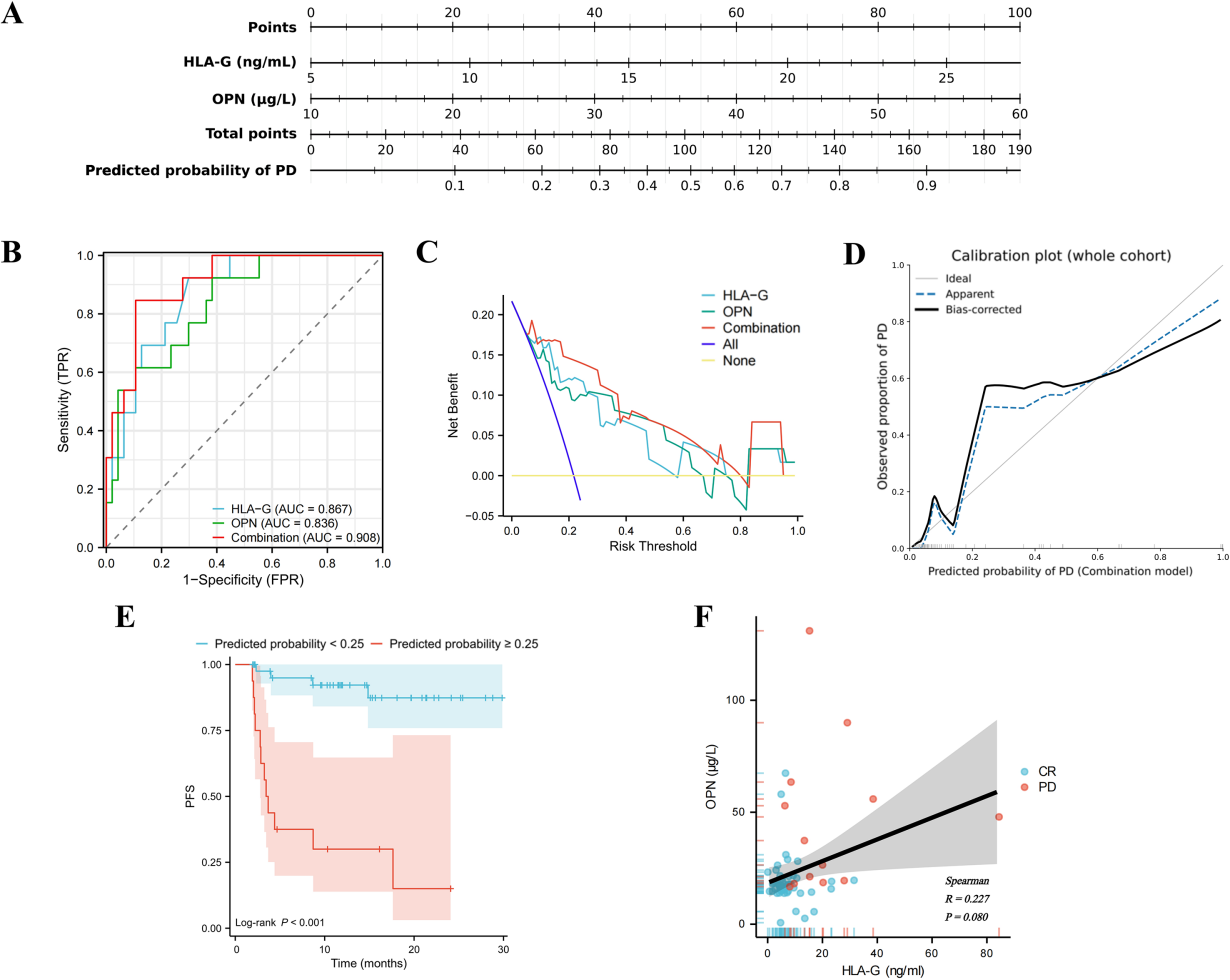
Exploratory development and internal assessment of the HLA-G–OPN two-marker model. **(A)** Nomogram for estimating the probability of progressive disease (PD) based on pretreatment plasma human leukocyte antigen G (HLA-G; ng/mL) and osteopontin (OPN; μg/L). Points assigned to each biomarker are summed to obtain total points, which correspond to the predicted probability of PD. The nomogram is exploratory and is not intended for clinical use without external validation. **(B)** Apparent receiver operating characteristic curves comparing discrimination of HLA-G alone, OPN alone and the combined model for predicting PD in the full cohort. Area under the curve values are indicated. **(C)** Exploratory decision curve analysis in the development cohort showing net benefit of prediction strategies across threshold probabilities, including HLA-G alone, OPN alone and the combined model, compared with treat-all and treat-none strategies. **(D)** Calibration plot of the combined model in the full cohort, showing apparent performance and bootstrap bias-corrected calibration against the ideal line. **(E)** Kaplan–Meier analysis of progression-free survival (PFS) stratified by predicted probability of PD derived from the combined model (cohort-derived cutoff of 0.25). Shaded areas denote 95% confidence intervals; two-sided log-rank P value is shown. **(F)** Scatter plot showing the relationship between pretreatment plasma HLA-G and OPN levels in the full cohort, colored by treatment outcome (complete response (CR) versus PD), with Spearman rank correlation (two-sided) and 95% confidence band. All model-development and performance analyses were conducted in the same full cohort of 60 patients, including 13 PD events. Bootstrap resampling provided internal optimism correction; no independent external validation was performed. The cutoff of 0.25 was derived from this cohort and should be considered exploratory. HLA-G, human leukocyte antigen G; OPN, osteopontin; PD, progressive disease; CR, complete response; ROC, receiver operating characteristic; PFS, progression-free survival; AUC, area under the curve.

We developed a two-marker logistic model combining pretreatment plasma HLA-G and OPN in the full cohort of 60 patients, including 13 PD events (**Supplementary Table 6**; **Figure 5A**). The model showed an apparent AUC of 0.908 (95% CI, 0.816–0.974), compared with 0.867 for HLA-G and 0.836 for OPN alone; bootstrap optimism correction yielded an AUC of 0.898 (**Figure 5B**; **Table 2**).

**Table 2:** Exploratory performance of ELISA biomarkers and the two-marker model in the full cohort.

Metric	HLA-G	OPN (SPP1)	Two-marker model
AUC (95% CI)^1^	0.867 (0.754–0.952)	0.836 (0.719–0.938)	0.908 (0.816–0.974)
Cutoff^2^	8.085 (ng/mL)	18.110 (μg/L)	0.250 (probability)
Sensitivity^3^	92.3%	92.3%	84.6%
Specificity^3^	70.2%	61.7%	89.4%
Optimism-corrected AUC^4^	0.863	0.834	0.898
PPV^5^	46.1%	40.0%	68.8%
NPV^5^	97.1%	96.7%	95.5%
Balanced accuracy^6^	81.2%	77.0%	87.0%
Continuous NRI (95% CI)^7^ (Combination vs HLA-G)	_	_	1.007 (0.400–1.556)
Continuous NRI (95% CI)^7^ (Combination vs OPN)	_	_	1.085 (0.523–1.601)

^1^ AUC 95% confidence intervals were calculated by bootstrap resampling (n=1000; percentile method).

^2^ The optimal cutoff was derived from the same cohort using the Youden index (sensitivity + specificity − 1); for the combination logistic model, the cutoff is on the predicted probability scale.

^3^ Sensitivity and specificity are reported at the selected cutoff. Sensitivity and specificity are apparent estimates calculated in the model-development cohort.

^4^ Optimism-corrected AUC denotes the bootstrap optimism-corrected AUC (internal bootstrap assessment; n=1000).

^5^ Positive and negative predictive values (PPV/NPV) depend on outcome prevalence; values shown were calculated assuming the observed PD prevalence in this cohort (13/60, 21.7%) using Bayes’ theorem: PPV = (Se×Prev)/(Se×Prev + (1−Sp)×(1−Prev)); NPV = (Sp×(1−Prev))/((1−Se)×Prev + Sp×(1−Prev)). To project PPV/NPV to other settings, substitute the expected prevalence (Prev).

^6^ BA (balanced accuracy) was defined as (sensitivity + specificity)/2 to account for class imbalance.

^7^ Continuous NRI (category-free NRI) quantifies net reclassification improvement of the combination model versus the corresponding single-marker logistic model (HLA-G or OPN), estimated by bootstrap resampling (n=1000).

PD, progressive disease; OPN, osteopontin; AUC, area under the curve; NRI, net reclassification improvement; PPV, positive predictive value; NPV, negative predictive value; Se, sensitivity; Sp, specificity; Prev, prevalence.

A cohort-derived cutoff of 0.25 yielded a sensitivity of 84.6%, specificity of 89.4%, positive predictive value of 68.8%, and negative predictive value of 95.5%. Calibration, continuous NRI, decision-curve, and PFS-stratification analyses are shown in **Figures 5C–E** and **Table 2**. HLA-G and OPN were weakly and non-significantly correlated (Spearman ρ = 0.227, P = 0.080), suggesting that they may capture partially non-overlapping information (**Figure 5F**).

Because biomarker selection, coefficient estimation, cutoff derivation, and performance assessment were conducted in the same small cohort, all model-performance estimates are exploratory and may remain optimistic.

### 3.6 Exploratory tissue profiling identifies associations with CD8+ T-cell and CD163+ macrophage densities

**Figure 6:**
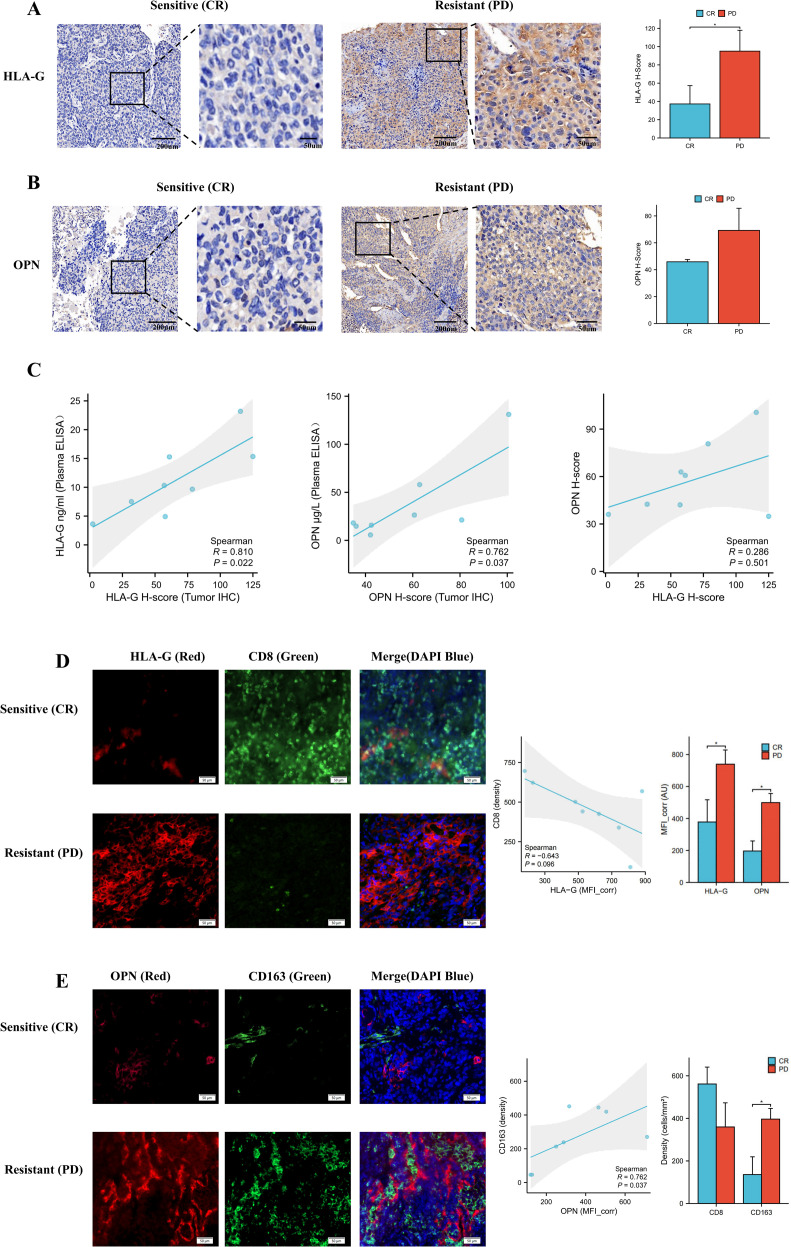
Exploratory tissue analysis of HLA-G, OPN, CD8+ T cells, and CD163+ macrophages in choriocarcinoma. **(A, B)** Representative immunohistochemistry images showing tumor expression of human leukocyte antigen G (HLA-G) **(A)** and osteopontin (OPN) **(B)** in choriocarcinoma tissues from patients with complete response (CR; PD-1 inhibitor–sensitive) or progressive disease (PD; PD-1 inhibitor–resistant), with corresponding H-score quantification shown at right. H-scores varied across cases, consistent with non-homogeneous expression. Boxed regions indicate areas magnified in the adjacent panels. **(C)** Correlations between plasma biomarker levels measured by enzyme-linked immunosorbent assay (ELISA) and tissue H-scores for HLA-G (left) and OPN (middle), and between tissue HLA-G and OPN H-scores (right). Spearman rank correlation coefficients (two-sided) and P values are shown; shaded areas indicate 95% confidence intervals. **(D)** Dual immunofluorescence showing HLA-G (red) and CD8 (green) with 4′, 6-diamidino-2-phenylindole (DAPI; blue) nuclear counterstain in representative CR and PD tumors, with quantification of CD8+ cell density versus HLA-G signal (scatter, left) and group comparisons of mean fluorescence intensity (MFI) for HLA-G and OPN (right). **(E)** Dual immunofluorescence showing OPN (red) and CD163 (green) with DAPI (blue) in representative CR and PD tumors, with correlation between CD163+ cell density and OPN signal (scatter, left) and group comparisons of CD8 and CD163 cell densities (right). For panels **(A, B, D)** and **(E)**, group comparisons (CR vs PD) were assessed using the Mann–Whitney U test (two-sided). For panels **(C–E)** (scatter plots), associations were evaluated using Spearman’s rank correlation (two-sided). *Significance is denoted as P < 0.05. Scale bars, as indicated. CR, complete response; PD, progressive disease; HLA-G, human leukocyte antigen G; OPN, osteopontin; ELISA, enzyme-linked immunosorbent assay; DAPI, 4′, 6-diamidino-2-phenylindole; MFI, mean fluorescence intensity.

Archival surgical tissues were available from eight patients (4 CR and 4 PD). Their baseline characteristics are summarized in **Supplementary Tables 7** and **S11**. Given the small and selected sample, this subset cannot be assumed to be representative of the full cohort. In this limited tissue subset, IHC showed higher HLA-G protein expression in PD tumors, with significantly increased H-scores in PD (P = 0.029) (**Figure 6A**; **Supplementary Table 8**). OPN also showed higher expression by IHC in PD, although the difference was less pronounced in this limited tissue set (**Figure 6B**; **Supplementary Table 8**).

Importantly, plasma levels of both biomarkers were positively correlated with their corresponding tissue measurements in this limited subset: plasma HLA-G correlated positively with tumor HLA-G H-scores, and plasma OPN correlated with tumor OPN H-scores (**Figure 6C**). These findings suggest concordance between circulating and tissue measurements but do not establish the cellular or tissue origin of the circulating proteins. By contrast, tumor HLA-G and OPN expression were not strongly correlated with each other (**Figure 6C**), suggesting that they may reflect partially distinct microenvironmental programs.

Exploratory duplex IF was used to examine immune-cell densities in the evaluated CR and PD specimens (**Figures 6D, E**). First, in the limited tissue subset, higher HLA-G intensity was accompanied by a trend toward lower CD8+ T-cell density; however, this association did not reach conventional statistical significance (**Figure 6D**). Second, higher OPN intensity was associated with greater CD163+ macrophage density in the evaluated specimens (P = 0.037), with higher CD163+ density in PD compared with CR (P = 0.030) (**Figure 6E**; **Supplementary Table 8**). Given the small tissue sample and absence of perturbation experiments, these associations should be considered hypothesis-generating. Case-level quantitative data are provided in **Supplementary Table 9**. To address spatial heterogeneity, CD8+ T-cell and CD163+ macrophage densities were additionally quantified separately in intratumoral and peritumoral compartments, as summarized in **Supplementary Table 13**.

### 3.7 Public-dataset analyses provide supportive biological context

**Figure 7:**
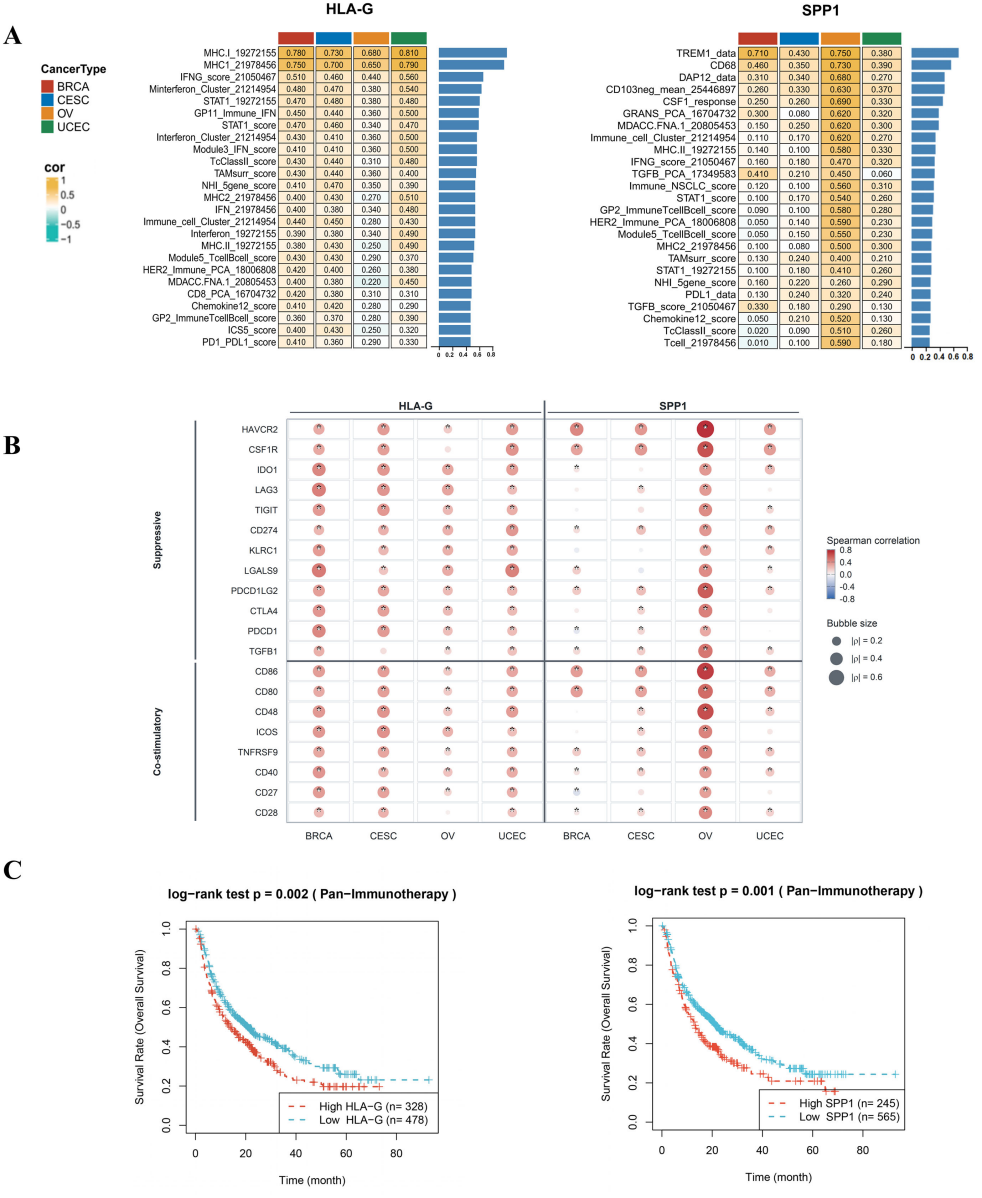
Public-dataset analyses show associations of HLA-G and SPP1 expression with immune-related transcriptional features and survival. **(A)** Associations between human leukocyte antigen G (HLA-G) or secreted phosphoprotein 1 (SPP1; osteopontin) expression and published immune signature scores across gynecologic cancer types, including breast invasive carcinoma (BRCA), cervical squamous cell carcinoma and endocervical adenocarcinoma (CESC), ovarian serous cystadenocarcinoma (OV) and uterine corpus endometrial carcinoma (UCEC). Heatmap values indicate correlation coefficients, and bar plots summarize the correlation magnitude across signatures. **(B)** Bubble heatmap showing Spearman correlations between HLA-G or SPP1 expression and selected immunomodulatory genes within BRCA, CESC, OV, and UCEC. Genes are grouped as suppressive or co-stimulatory and ordered within each group according to the median absolute correlation across the eight marker–cancer combinations. Bubble color indicates the direction and magnitude of the Spearman correlation coefficient, bubble size indicates |ρ|, and asterisks denote Benjamini–Hochberg-adjusted FDR <0.05. These transcriptomic co-expression patterns do not imply direct functional interactions. **(C)** Kaplan–Meier analyses of overall survival in a pan-immunotherapy cohort stratified by high versus low expression of HLA-G (left) or SPP1 (right). Two-sided log-rank P values are shown; numbers in parentheses indicate sample sizes in each group. *HLA-G, human leukocyte antigen G; SPP1, secreted phosphoprotein 1 (osteopontin); BRCA, breast invasive carcinoma; CESC, cervical squamous cell carcinoma and endocervical adenocarcinoma; OV, ovarian serous cystadenocarcinoma; UCEC, uterine corpus endometrial carcinoma.*.

To place the HLA-G and SPP1/OPN findings in a broader immunologic context, we analyzed public transcriptomic datasets from gynecologic cancers and immunotherapy-treated cohorts. Using TCGA transcriptomic data, we focused on four gynecologic cancer types (BRCA, CESC, OV, and UCEC). Across these cancers, HLA-G primarily aligned with IFN/STAT1-driven antigen-presentation and checkpoint activity signatures, whereas SPP1 preferentially tracked myeloid–macrophage programs (e.g., TREM1/DAP12 and CSF1R/CD68-related scores) (**Figure 7A**), broadly consistent with the exploratory tissue associations observed in our cohort.

We next examined correlations between HLA-G or SPP1 expression and selected immunomodulatory genes within each cancer type. HLA-G showed broadly positive correlations with multiple suppressive and co-stimulatory genes across the four cancer types. In contrast, correlations involving SPP1 were more heterogeneous across cancer types and were most pronounced in OV (**Figure 7B**). These findings describe cancer-type-dependent transcriptomic co-expression patterns and do not imply direct functional interactions.

To assess potential clinical relevance under immune checkpoint blockade, we analyzed a public pan-immunotherapy cohort and found that high expression of either HLA-G or SPP1 was associated with significantly worse overall survival (HLA-G: log-rank P = 0.002; SPP1: log-rank P = 0.001) (**Figure 7C**). Together, these cross-cancer analyses provide supportive biological context for associations of HLA-G and SPP1/OPN expression with immune-related transcriptional programs and unfavorable outcomes in immunotherapy-treated populations. However, because these datasets involved different tumor types, tissue RNA expression rather than circulating protein measurements, and different clinical endpoints, they do not constitute external validation of the plasma biomarkers in choriocarcinoma.

## 4 Discussion

### 4.1 Principal findings and clinical context

In this study, elevated pretreatment plasma HLA-G and OPN were associated with primary resistance to PD-1 inhibitor plus chemotherapy in high-risk choriocarcinoma. Their combination showed promising but exploratory discrimination within the development cohort. Limited paired-tissue analyses further suggested that HLA-G and OPN may reflect partially distinct immune microenvironmental features, although these associations require independent and functional validation.

Despite the high activity of PD-1 inhibitor–based combinations in refractory GTN, a subset of patients remains primarily resistant. Because PD-L1 is widely expressed in trophoblastic tumors but has limited discriminatory value (11, 12), plasma HLA-G and OPN warrant further evaluation as candidate pretreatment markers.

### 4.2 HLA-G and its potential association with CD8-poor immune states

HLA-G is a non-classical MHC-I molecule physiologically enriched at the maternal–fetal interface, where it contributes to immune tolerance by restraining NK and T-cell effector functions (13, 26). Mechanistically, HLA-G signals through inhibitory receptors including ILT2, ILT4, and KIR2DL4 (15, 27), enabling broad suppression across innate and adaptive compartments. Experimental evidence shows that HLA-G can directly suppress CD8+ CTL function (15); soluble HLA-G may also induce apoptosis in NK and CD8+T cell (28, 29). These tolerogenic properties are indispensable for pregnancy, yet tumors can co-opt them as an immune escape program, supporting the concept of HLA-G as a non-classical immune checkpoint beyond PD-1/PD-L1 (30).

Consistent with this biology, higher tumor HLA-G expression was accompanied by a non-significant trend toward lower CD8+ T-cell density in our limited tissue subset. Notably, recent work also indicates that HLA-G can shape multiple additional suppressive axes—including impairing dendritic cell activation/antigen presentation (30). Circulating HLA-G may therefore reflect aspects of a tissue immune-tolerance state, although this interpretation requires confirmation in larger paired plasma–tissue cohorts and functional studies.

### 4.3 OPN/SPP1 and its association with CD163+ macrophage enrichment

Osteopontin (OPN, encoded by SPP1) is a multifunctional secreted phosphoprotein that can function as a chemotactic factor for macrophages and neutrophils (16). Emerging work has framed OPN signaling as an immune-regulatory axis that can suppress antitumor immunity (18, 31). It has been linked to macrophage recruitment, M2-like polarization, and suppression of CD8+ T-cell activity through CD44- and integrin-dependent pathways (16, 19). Clinical and translational datasets further indicate that high SPP1 programs are associated with macrophage-rich suppressive ecosystems and dysfunctional T-cell states (18, 31–34).

In our exploratory tissue subset, the observed association was consistent with this biological hypothesis: higher OPN intensity was associated with greater CD163+ macrophage density in the evaluated specimens, suggesting a possible relationship between OPN expression and a CD163+ macrophage-enriched tissue state that may limit effective PD-1–mediated T-cell reinvigoration. Thus, HLA-G and OPN may be associated with partially distinct tissue immune patterns—lower CD8+ T-cell density and greater CD163+ macrophage density, respectively.

### 4.4 Complementary biomarker information and potential clinical implications

HLA-G and OPN were only weakly correlated, suggesting that they may capture partially non-overlapping biological information and providing a rationale for evaluating their combined biomarker performance. Neither biomarker showed a strong correlation with FIGO prognostic score, and their associations with PD remained directionally consistent after separate adjustment for selected clinical variables. However, these limited analyses do not establish independence from tumor burden, treatment regimen, prior treatment exposure, or systemic inflammation, and residual confounding remains possible.

In choriocarcinoma, treatment is predominantly systemic, and pretreatment tumor tissue is often unavailable because surgery is generally reserved for selected indications (1, 2). Plasma-based biomarkers may therefore be more feasible than tissue-based markers for pretreatment assessment. In the small paired plasma–tissue subset, circulating HLA-G and OPN levels correlated with their corresponding tissue measurements and showed exploratory associations with immune-cell densities. Nevertheless, the apparent improvement in discrimination achieved by the combined model was observed within the same development cohort and does not establish clinical utility. If independently validated, plasma HLA-G and OPN may provide a minimally invasive approach for pretreatment risk assessment. Prospective multicenter studies are required to determine whether these biomarkers add predictive value beyond established clinical factors and whether biomarker-guided monitoring or treatment adaptation improves clinical outcomes.

Notably, public transcriptomic analyses showed recurring associations of HLA-G and SPP1 with immune-related gene-expression programs and unfavorable outcomes in immunotherapy-treated populations. These findings provide supportive biological context but should not be interpreted as validation of the circulating protein biomarkers.

The context of chemotherapy plus immunotherapy introduces a paradox. While chemotherapy is intended to induce immunogenic cell death, it can act as a “double-edged sword” by altering the tumor microenvironment (35). Cytotoxic stress induces the release of inflammatory cytokines, such as IL-6, which can paradoxically upregulate immune checkpoints and promote resistance (36). Although our ELISA results for IL-6R were not statistically significant, our proteomic data indicated an enrichment of inflammatory response pathways in resistant patients. One hypothesis raised by prior studies is that inflammatory signaling, including STAT3-related pathways, could regulate HLA-G and OPN expression (18, 30, 31, 37). However, treatment-induced changes and STAT3 pathway activity were not evaluated in the present study, and this possibility remains speculative. This provides hypothesis-generating biological context for the observed association between pretreatment HLA-G/OPN levels and early resistance.

### 4.5 Potential translational hypotheses involving HLA-G/ILT and SPP1-associated myeloid pathways

The associations of HLA-G and OPN with treatment resistance raise testable, but currently unproven, hypotheses for biomarker-guided combination strategies. HLA-G and its inhibitory receptors ILT2 and ILT4 represent potential therapeutic targets (30). Early-phase studies are evaluating HLA-G blockade with TTX-080, ILT4 inhibition with MK-4830, and dual ILT2/ILT4 antagonism with NGM707, including combinations with PD-1 blockade (38–43). On the myeloid side, SPP1/OPN-associated immunosuppressive programs have been linked to macrophage-rich tumor microenvironments and dysfunctional antitumor immunity (18, 31–34). These programs may potentially be addressed through disruption of OPN–CD44 signaling or modulation of the CSF1R axis (17, 44).

However, these therapeutic approaches have not been evaluated in choriocarcinoma, and the associative findings of the present study do not support biomarker-directed treatment selection. Functional perturbation studies and prospective clinical evaluation will be required to determine whether targeting HLA-G/ILT or SPP1-associated myeloid pathways can overcome resistance to PD-1 inhibitor–based therapy.

### 4.6 Limitations and future directions

This study has several limitations. First, the study was limited by the rarity of choriocarcinoma, with only 60 patients and 13 PD events. The 30-patient proteomic discovery subset was nested within the full cohort used for ELISA assessment, and biomarker selection, model fitting, and performance evaluation were all conducted in the same dataset. Although bootstrap resampling and sensitivity analyses were used for internal assessment, they cannot substitute for independent validation. Therefore, the biomarker associations and model performance remain exploratory and require confirmation in independent multicenter cohorts. In addition, although the global enrichment analyses were not restricted by immune-related prefiltering, the subsequent immune-focused candidate-prioritization strategy may have overlooked informative non-immune biomarkers. Second, the tissue analyses were exploratory and based on only eight surgical specimens. No perturbation experiments, including HLA-G or OPN knockdown, T-cell activation assays, or macrophage polarization assays, were performed. Therefore, these correlation-based observations do not establish that HLA-G or OPN directly mediates resistance and require dedicated functional validation. Third, residual clinical confounding remains possible. Because only 13 PD events were available, all potential confounders could not be adjusted for simultaneously. Although separate limited-adjustment analyses yielded directionally consistent biomarker associations, they do not establish independence from tumor burden, metastatic distribution, or regimen selection. In addition, inflammatory and infectious-status variables were not uniformly available.

In summary, elevated circulating HLA-G and OPN were associated with primary resistance to PD-1 inhibitor plus chemotherapy in high-risk choriocarcinoma and may reflect distinct tumor immune microenvironmental states. These candidate biomarkers and the exploratory two-marker model may provide a preliminary framework for future pretreatment risk-stratification research, but require independent clinical validation and functional investigation.

## Data Availability

The datasets presented in this study can be found in online repositories. The names of the repository/repositories and accession number(s) can be found below: http://www.proteomexchange.org/, PXD074920.
