# Supplementary material for: Integrated plasma proteomics identifies HLA-G and osteopontin as circulating biomarkers of resistance to PD-1 inhibitor plus chemotherapy in choriocarcinoma
Source: Front Immunol. 2026 Aug 4;17:1817862. doi: 10.3389/fimmu.2026.1817862 (PMC13481723; doi:10.3389/fimmu.2026.1817862)
Supplement: Supplementary file 1 [file Table1.docx]

**Supplementary Methods**

**Supplementary Methods S1. Response assessment and definition of CR and PD**

This supplementary section provides detailed definitions and operational criteria for treatment response classification in patients with high-risk choriocarcinoma treated with PD-1 inhibitor plus chemotherapy. Response evaluation integrated radiologic assessment (RECIST v1.1) and serial serum β-hCG monitoring.

1. **Radiologic assessment (RECIST v1.1)**

Baseline imaging was obtained prior to treatment initiation. Cross-sectional imaging (contrast-enhanced CT and/or MRI; with brain MRI when clinically indicated) was repeated at protocol-defined intervals (every 6–8 weeks or every 2 cycles) and at any time progression was clinically suspected. Target lesions were selected at baseline according to RECIST version 1.1. Radiologic response categories (CR/PR/SD/PD) were determined based on (i) changes in the sum of diameters of target lesions, (ii) the status of non-target lesions, and (iii) the appearance of new lesions. When feasible, radiologic evaluations were reviewed by investigators and independent radiologists blinded to biomarker measurements.

2. **Serologic assessment (serum β-hCG)**

Serum β-hCG was measured at baseline and serially during treatment using a standardized clinical assay in the institutional laboratory. Testing was performed according to institutional practice (weekly during active treatment and at follow-up visits). To ensure analytical consistency, all measurements were reported in IU/L and interpreted using the institutional reference range for the assay.

Definitions used for serologic response/progression were as follows:

- Serologic complete response (serologic CR): normalization of serum β-hCG to within the institutional reference range (< 5IU/L), confirmed by at least two consecutive measurements obtained on separate days (≥7 days apart).
- Serologic progression (serologic PD): any of the following after treatment initiation, confirmed on repeat testing when applicable: (i) re-elevation of β-hCG above the upper limit of normal after prior normalization; (ii) sustained rise in β-hCG relative to the nadir (an increase on ≥2 consecutive assessments); or (iii) plateau pattern consistent with biochemical non-response, defined as minimal change (within ±10%) across serial measurements over a clinically meaningful interval ( ≥3 weeks).

3. **Composite outcome definition for CR and PD groups**

For biomarker analyses, patients were classified into two clinical outcome groups using a composite rule integrating radiologic and serologic evaluations:

- Clinical complete response (CR): normalization of serum β-hCG (confirmed as defined) with no radiologic evidence of progressive disease per RECIST v1.1 (radiologic CR/PR/SD, but not PD), and no clinical evidence of progression during the on-treatment assessment period. Because post-treatment residual lesions may represent fibrosis/necrosis rather than viable tumor in choriocarcinoma, β-hCG normalization was prioritized as the key criterion for complete response, while radiologic assessment was used primarily to exclude progression (RECIST v1.1 PD).
- Clinical progressive disease (PD): occurrence of radiologic PD by RECIST v1.1 and/or serologic PD as defined above. If radiologic and serologic assessments were discordant, PD was assigned when either criterion for progression was met.

4. **Timing definitions and survival endpoints**

The date of best response was defined as the first date on which CR criteria were met. Progression was dated as the earliest time point when progression (radiologic or serologic) was first documented (and subsequently confirmed when required). Progression-free survival (PFS) was calculated from treatment initiation to the first documented progression event or last follow-up, whichever occurred first. Overall survival (OS), was calculated from treatment initiation to death from any cause or last follow-up.

5. **Quality control and handling of ambiguous assessments**

When response determination was uncertain, cases were adjudicated by consensus review among treating investigators, using all available longitudinal clinical, imaging, and laboratory information. If multiple imaging modalities were available, the modality providing the most comprehensive assessment of measurable disease burden was prioritized.

6. **Abbreviations**

- β-hCG, beta-human chorionic gonadotropin
- CR, complete response
- CT, computed tomography
- MRI, magnetic resonance imaging
- OS, overall survival
- PD, progressive disease
- PFS, progression-free survival
- PR, partial response
- RECIST, Response Evaluation Criteria in Solid Tumors
- SD, stable disease

**Supplementary Table S2. ELISA assay characteristics for plasma biomarker quantification**

| **Analyte** | **HLA-G (MHCG/HLA-G)** | **CXCL12 / SDF-1α** | **OPN / SPP1 (Osteopontin)** | **PD-L1 / CD274** | **IL-6R (Interleukin-6 receptor)** |
| --- | --- | --- | --- | --- | --- |
| **Manufacturer** | Elabscience | Abcam | Bioswamp | Bioswamp | Bioswamp |
| **Catalog No.** | E-EL-H1663 | ab100637 | HM10043 | HM10627 | HM10449 |
| **Assay format** | Sandwich ELISA | Sandwich ELISA | Sandwich ELISA | Sandwich ELISA | Sandwich ELISA |
| **Validated matrices (per kit)** | Human serum; plasma (EDTA); other biological fluids | Human serum/plasma; cell culture supernatant | Human serum; plasma; other biological fluids | Human serum; plasma; other biological fluids | Human serum; plasma; other biological fluids |
| **Report unit** | ng/mL | pg/mL | μg/L | pg/mL | ng/L |
| **Standard curve range** | 0.63–40 | 93.75–6000 | 0.8–40 | 10–360 | 15–400 |
| **Sensitivity / LOD** | 0.38 ng/mL | 42 pg/mL | Not specified in booklet | Not specified in booklet | Not specified in booklet |
| **Precision (intra-assay CV)** | 6.25–6.94% | <10% | <9% | <9% | <9% |
| **Precision (inter-assay CV)** | 6.89–8.90% | <12% | <15% | <15% | <15% |
| **Standard levels (example series)** | 40, 20, 10, 5, 2.5, 1.25, 0.63, 0 | 6000, 3000, 1500, 750, 375, 187.5, 93.75, 0 | 30, 20, 10, 5, 2.5 (plus 0) | 360, 240, 120, 60, 30 (plus 0); standard stock 540 pg/mL | 360, 240, 120, 60, 30 (plus 0); standard stock 540 ng/L |
| **Key storage / shelf-life** | Store at 2–8 °C; shelf-life 12 months (unopened) | Store at 2–8 °C; shelf-life 6 months | Store at 2–8 °C; shelf-life 6 months | Store at 2–8 °C; shelf-life 6 months | Store at 2–8 °C; shelf-life 6 months |
| **Readout** | OD450 nm | OD450 nm | OD450 nm | OD450 nm | OD450 nm |
| **Notes** | If OD exceeds upper standard, dilute and multiply by dilution factor. | Standard curve typically fit with 4-parameter logistic model. | Concentrations calculated from standard curve and multiplied by dilution factor. | Concentrations calculated from standard curve and multiplied by dilution factor. | Concentrations calculated from standard curve and multiplied by dilution factor. |

*Note:Samples above the upper limit of the standard curve were serially diluted ( 1:2–1:20 as needed) and re-assayed; reported concentrations were back-calculated by multiplying the dilution factor.*

**Supplementary Methods S3. Public database analyses (TCGA and pan-immunotherapy)**

1. **Data sources and cohort definition**

We interrogated transcriptomic and immune-related features in four gynecologic cancer types (BRCA, CESC, OV, and UCEC) from the TCGA Pan-Cancer Atlas resource. TCGA RNA-seq expression data were obtained from the UCSC Xena browser as the Pan-Cancer Atlas batch-corrected expression matrix (EBPlusPlusAdjustPANCAN_IlluminaHiSeq_RNASeqV2.geneExp.tsv). Curated clinical annotations and survival endpoints were taken from the TCGA Pan-Cancer Clinical Data Resource (TCGA-CDR) and accessed via the UCSC Xena dataset “phenotype – Curated clinical data”. Expression and clinical tables were matched by TCGA sample identifiers, and only samples present in both datasets were retained. When multiple aliquots were available for a given case, a single representative primary tumor sample was retained according to UCSC Xena/TCGA standard sample identifiers.

2. **Pre-processing**

TCGA expression values were used as provided by UCSC Xena (log2-scale where applicable). For analyses requiring log2-scale input, values were log2-transformed as log2(x + 1) if needed. For cross-cohort analyses (pan-immunotherapy), gene expression was Z-score standardized within each cohort to reduce between-study platform effects. Unless otherwise specified, extreme outliers were excluded after within-cohort Z-scoring (|Z| > 3 for the analyzed gene), and the remaining samples were carried forward. For survival analyses, cases with missing survival time/status or survival time equal to 0 were excluded.

3. **Immune signature association analyses (TCGA gynecologic cancers)**

To evaluate immune associations of the target genes (HLA-G and SPP1/OPN) within BRCA, CESC, OV, and UCEC, a compendium of published immune expression signatures (68 signatures) was obtained from UCSC Xena. For each cancer type, Spearman rank correlations (two-sided) were computed between target gene expression and each immune signature score (cor.test in R). Correlation matrices were visualized as heatmaps. P values were adjusted for multiple testing using the Benjamini–Hochberg method within each cancer type/signature set.

4. **Immune checkpoint gene association analyses (TCGA gynecologic cancers)**

To summarize correlations between the target genes and immune checkpoint–related genes within BRCA, CESC, OV, and UCEC, immune checkpoint gene lists (immunoinhibitors and immunostimulators) were retrieved from TISIDB. For each cancer type, Spearman correlation coefficients were computed between target gene expression and each checkpoint gene across samples. P values were adjusted using the Benjamini–Hochberg method within each marker–cancer analysis. For the main-text visualization, selected genes were grouped as suppressive or co-stimulatory and ordered within each group according to the median absolute correlation across the eight marker–cancer combinations. Bubble color represents the Spearman correlation coefficient, bubble size represents |ρ|, and asterisks indicate FDR <0.05.

5. **Pan-immunotherapy survival analyses**

To assess the prognostic relevance of checkpoint-related genes under immune checkpoint blockade (ICB), we analyzed a pooled “pan-immunotherapy” dataset curated in the TIDE framework. In brief, ICB cohorts were stratified by treatment stage, gene expression values were z-scored within each cohort/stage, and then combined after removing outliers. For time-to-event analyses, an outcome-oriented cutoff was determined using maximally selected log-rank statistics (selecting the cutpoint that maximized the log-rank statistic / minimized the log-rank P value) within the analyzed cohort. This cutoff was then used to dichotomize samples into high and low expression groups. Overall survival was compared between high- and low-expression groups using Kaplan–Meier curves and the log-rank test.

6. **Included pretreatment ICB cohorts (overall survival)**

Dataset name

SKCM_TILs_GSE100797_ACT_Pre

SKCM_Tumor_GSE78220_anti-PD1_Pre

SKCM_Tumor_PMID31792460_anti-PD1_Pre

SKCM_Tumor_PMID27956380_anti-CTLA4_Pre

SKCM_Tumor_PMID30753825_anti-PD1_Pre

SKCM_Tumor_PMID30753825_anti-PD1-CTLA4_Pre

BLCA_Tumor_IMvigor210_anti-PD1_Pre

BLCA_Tumor_GSE176307_anti-PD1_Pre

KIRC_Tumor_PMID32472114_anti-PD1_Pre

7. **Software**

All analyses were performed in R (version as specified in the main Methods). Core packages included survival and survminer for Kaplan–Meier analyses, ComplexHeatmap for visualization, and standard tidyverse utilities for data wrangling.

8. **Abbreviations**

- BRCA, breast invasive carcinoma
- CESC, cervical squamous cell carcinoma and endocervical adenocarcinoma;
- OV, ovarian serous cystadenocarcinoma;
- UCEC, uterine corpus endometrial carcinoma;
- TCGA, The Cancer Genome Atlas
- ICB, immune checkpoint blockade.
- TISIDB, Tumor-Immune System Interaction Database
- TIDE, Tumor Immune Dysfunction and Exclusion

9. **Supplementary references**
1. Weinstein JN, Collisson EA, Mills GB, et al. The Cancer Genome Atlas Pan-Cancer analysis project. Nat Genet. 2013;45(10):1113–1120.
2. Liu J, Lichtenberg T, Hoadley KA, et al. An integrated TCGA Pan-Cancer clinical data resource to drive high-quality survival outcome analytics. Cell. 2018;173(2):400–416.e11.
3. Jiang P, Gu S, Pan D, et al. Signatures of T cell dysfunction and exclusion predict cancer immunotherapy response. Nat Med. 2018;24(10):1550–1558.
4. Amara D, Wolf DM, van ’t Veer L, Esserman L, Campbell M, Yau C. Co-expression modules identified from published immune signatures reveal five distinct immune subtypes in breast cancer. Breast Cancer Res Treat. 2017;161(1):41–50.
5. Ru B, Wong CN, Tong Y, et al. TISIDB: an integrated repository portal for tumor–immune system interactions. Bioinformatics. 2019;35:4200–4202.
6. Goldman MJ, Craft B, Hastie M, et al. Visualizing and interpreting cancer genomics data via the UCSC Xena platform. Nat Biotechnol. 2020;38:675–678.
