# Supplementary material for: Integrated plasma proteomics identifies HLA-G and osteopontin as circulating biomarkers of resistance to PD-1 inhibitor plus chemotherapy in choriocarcinoma
Source: Front Immunol. 2026 Aug 4;17:1817862. doi: 10.3389/fimmu.2026.1817862 (PMC13481723; doi:10.3389/fimmu.2026.1817862)
Supplement: Supplementary file 2 [file Table2.docx]

**`Supplementary Table S1. Baseline characteristics of the nested proteomic discovery subset (n = 30), stratified by treatment outcome.**

| **Characteristic** | **CR (n=21)** | **PD (n=9)** | **P value** |
| --- | --- | --- | --- |
| Treatment status |  |  | 1.000 |
| Primary | 15 (71.4%) | 7 (77.8%) |  |
| Recurrent | 6 (28.6%) | 2 (22.2%) |  |
| FIGO stage |  |  | 0.185 |
| I | 1 (4.8%) | 1 (11.1%) |  |
| II | 0 (0.0%) | 1 (11.1%) |  |
| III | 12 (57.1%) | 2 (22.2%) |  |
| IV | 8 (38.1%) | 5 (55.6%) |  |
| FIGO prognostic score | 12.00 [10.00–13.00] | 14.00 [10.00–18.00] | 0.151 |
| Age, years | 32.00 [28.00–37.00] | 29.00 [26.00–30.00] | 0.195 |
| Interval from antecedent pregnancy to chemotherapy, months | 20.00 [10.00–40.00] | 20.00 [7.00–60.00] | 0.786 |
| Pretreatment β-hCG, IU/L | 57294.00 [2816.00–261830.00] | 113576.00 [2392.00–225000.00] | 0.651 |
| Largest tumor diameter, cm | 3.00 [1.90–7.00] | 6.00 [5.20–6.90] | 0.174 |
| Antecedent pregnancy |  |  | 0.881 |
| Abortion | 7 (33.3%) | 2 (22.2%) |  |
| Molar pregnancy | 7 (33.3%) | 3 (33.3%) |  |
| Term delivery | 7 (33.3%) | 4 (44.4%) |  |
| Pelvic metastasis |  |  | 0.622 |
| No | 18 (85.7%) | 7 (77.8%) |  |
| Yes | 3 (14.3%) | 2 (22.2%) |  |
| Lung metastasis |  |  | 0.143 |
| No | 2 (9.5%) | 3 (33.3%) |  |
| Yes | 19 (90.5%) | 6 (66.7%) |  |
| Brain metastasis |  |  | 0.389 |
| No | 16 (76.2%) | 5 (55.6%) |  |
| Yes | 5 (23.8%) | 4 (44.4%) |  |
| Liver metastasis |  |  | 0.622 |
| No | 18 (85.7%) | 7 (77.8%) |  |
| Yes | 3 (14.3%) | 2 (22.2%) |  |
| Other site metastasis |  |  | 0.563 |
| No | 19 (90.5%) | 7 (77.8%) |  |
| Yes | 2 (9.5%) | 2 (22.2%) |  |
| Number of metastatic lesions | 1.00 [1.00–2.00] | 3.00 [1.00–8.00] | 0.126 |
| History of chemotherapy failure |  |  | 0.443 |
| No | 13 (61.9%) | 4 (44.4%) |  |
| Yes | 8 (38.1%) | 5 (55.6%) |  |
| Chemotherapy regimen |  |  | 0.368 |
| EMA/CO | 9 (42.9%) | 3 (33.3%) |  |
| FAEV | 8 (38.1%) | 2 (22.2%) |  |
| Other | 4 (19.0%) | 4 (44.4%) |  |
| Immunotherapy agent |  |  | 0.431 |
| Camrelizumab | 15 (71.4%) | 5 (55.6%) |  |
| Toripalimab | 6 (28.6%) | 4 (44.4%) |  |
| Surgery performed |  |  | 0.691 |
| No | 11 (52.4%) | 6 (66.7%) |  |
| Yes | 10 (47.6%) | 3 (33.3%) |  |

*Note: Continuous variables are presented as median [interquartile range] and were compared using the Mann–Whitney U test. Categorical variables are presented as n (%) and were compared using Pearson’s chi-square test when appropriate; Fisher’s exact test was used for 2×2 tables with small expected counts. For r×c tables with sparse cells (e.g., FIGO stage, antecedent pregnancy, chemotherapy regimen), a Fisher–Freeman–Halton exact test was approximated by a permutation procedure (10,000 permutations).*

*Abbreviations: hCG, human chorionic gonadotropin; EMA/CO, etoposide–methotrexate–actinomycin D/cyclophosphamide–vincristine; FAEV, floxuridine–actinomycin D–etoposide–vincristine.*

**Supplementary Table S2. Immune response-related differentially expressed proteins (PD vs CR) in the nested proteomic discovery subset.**

| **Protein**  **(gene symbol)** | **logFC** | **AveExpr** | **t** | **P value** | **adj. P value** | **B** |
| --- | --- | --- | --- | --- | --- | --- |
| HLA-G | 3.945 | 21.687 | 8.216 | 2.11E-09 | 2.29E-07 | 11.540 |
| SPP1 | 3.043 | 23.190 | 6.921 | 7.58E-08 | 3.54E-06 | 8.035 |
| CXCL12 | 3.004 | 21.240 | 6.980 | 6.41E-08 | 3.34E-06 | 8.200 |
| CD274 | 1.705 | 25.497 | 6.557 | 2.14E-07 | 7.99E-06 | 7.019 |
| SAA1 | 1.384 | 25.284 | 6.830 | 9.80E-08 | 4.22E-06 | 7.783 |
| C5 | 1.062 | 19.098 | 5.943 | 1.26E-06 | 2.99E-05 | 5.284 |
| CRP | 1.055 | 22.626 | 5.840 | 1.69E-06 | 3.62E-05 | 4.992 |
| SAA2 | 1.046 | 19.590 | 5.393 | 6.24E-06 | 9.60E-05 | 3.718 |
| IL6R | 1.309 | 20.956 | 6.591 | 1.94E-07 | 7.57E-06 | 7.115 |
| ICAM3 | 1.375 | 18.389 | 5.435 | 5.52E-06 | 8.84E-05 | 3.837 |
| VCAM1 | 1.338 | 19.986 | 6.264 | 4.95E-07 | 1.54E-05 | 6.195 |
| TGFB1 | 1.021 | 21.014 | 5.531 | 4.17E-06 | 7.20E-05 | 4.111 |
| HLA-A | -2.552 | 22.790 | -9.636 | 5.30E-11 | 1.98E-08 | 15.133 |
| HLA-B | -2.203 | 20.670 | -9.404 | 9.52E-11 | 3.05E-08 | 14.564 |
| STAT1 | -1.257 | 18.036 | -5.940 | 1.27E-06 | 2.99E-05 | 5.276 |
| B2M | -2.023 | 22.138 | -7.485 | 1.56E-08 | 1.03E-06 | 9.586 |
| CXCL14 | -2.438 | 19.409 | -5.449 | 5.30E-06 | 8.62E-05 | 3.876 |

*Note: Differential expression was assessed using the limma framework. logFC, log2 fold change (PD vs CR); AveExpr, average log2 expression across all samples; t, moderated t-statistic; P value, raw P value; adj. P value, Benjamini–Hochberg false discovery rate (FDR)-adjusted P value; B, log-odds that the feature/protein is differentially expressed (limma B-statistic).*

**Supplementary Table S3. ELISA biomarkers by treatment outcome in the full cohort (N =60)**

| **Variable (ELISA)** | **CR (n=47)** | **PD (n=13)** | **P value** |
| --- | --- | --- | --- |
| HLA-G (ng/mL) | 6.00 [4.35–8.91] | 15.35 [9.67–27.93] | <0.001 |
| OPN (SPP1) (μg/L) | 17.49 [14.74–20.60] | 37.35 [19.74–55.90] | <0.001 |
| SDF-1 (CXCL12) (pg/mL) | 258.67 [92.00–375.33] | 147.56 [47.56–492.00] | 0.399 |
| IL-6R (ng/L) | 186.40 [167.05–265.29] | 201.52 [188.40–246.07] | 0.206 |
| PD-L1 (CD274) (pg/mL) | 191.23 [166.90–247.73] | 200.15 [186.79–310.24] | 0.156 |

*Data are presented as median [IQR]. P values were calculated using the two-sided Mann–Whitney U test. Analyses were based on available data (no imputation).*

*Abbreviations: CR, complete response; PD, progressive disease; ELISA, enzyme-linked immunosorbent assay; IQR, interquartile range.*

**Supplementary Table S4. Univariable and multivariable logistic regression of ELISA biomarkers (Z-scores) for PD vs CR**

| **Biomarker (per 1 SD increase; Z-score)** | **Univariable OR (95% CI)** | **P (univ)** | **Multivariable OR (95% CI)** | **P (multiv)** |
| --- | --- | --- | --- | --- |
| HLA-G ng/ml | 6.34 (2.00–20.07) | 0.002 | 7.07 (1.74–28.77) | 0.006 |
| OPN μg/L | 4.45 (1.68–11.75) | 0.003 | 4.41 (1.43–13.57) | 0.010 |
| SDF-1 pg/ml | 0.98 (0.53–1.83) | 0.960 | — | — |
| IL-6R ng/L | 1.05 (0.58–1.91) | 0.864 | — | — |
| PD-L1 pg/ml | 1.15 (0.65–2.01) | 0.635 | — | — |

*Note: Analyses were conducted in the full cohort of 60 patients, including the 30 patients used for proteomic discovery. All five proteins were standardized to Z-scores using the cohort mean and standard deviation (per 1 SD increase). Outcome was progressive disease (PD) versus complete response (CR). Univariable logistic regression was fitted separately for each biomarker. Biomarkers with univariable P<0.05 were entered into the multivariable logistic regression model. Odds ratios (ORs) and 95% confidence intervals (CIs) were derived from Wald statistics. P values were two-sided.*

**Supplementary Table S5. Integrated sensitivity analyses for HLA-G and OPN**

| **Strategy** | **Overview** | **Analysis** | **HLA-G** | **OPN** |
| --- | --- | --- | --- | --- |
| Strategy 1^1^ | Agent-controlled subgroup  (Camrelizumab-only) | Camrelizumab-only CR (median, IQR) | 5.50 (4.24–7.69) | 17.37 (14.68–20.60) |
|  |  | Camrelizumab-only PD (median, IQR) | 15.27 (10.96–24.55) | 47.91 (31.90–76.72) |
|  |  | P value | <0.01 | <0.01 |
| Strategy 2^2^ | Correlation with FIGO prognostic score | Spearman R  (vs FIGO score) | 0.103 | 0.064 |
|  |  | P value | 0.435 | 0.627 |
|  |  | N | 60 | 60 |
| Strategy 3^3^ | Limited adjustment logistic model (Model A)  ——adjust for immunotherapy agent | Model A: biomarker OR (95% CI) | 6.11 (1.83–20.33) | 5.34 (1.82–15.63) |
|  |  | P value | 0.003 | 0.002 |
|  |  | Model A: agent OR (95% CI) | 4.04 (0.84–19.53) | 8.14 (1.52–43.56) |
|  |  | P value | 0.082 | 0.014 |
|  | Limited adjustment logistic model (Model B)  ——adjust for FIGO prognostic score | Model B: biomarker OR (95% CI) | 5.80 (1.84–18.24) | 4.54 (1.66–12.44) |
|  |  | P value | 0.003 | 0.003 |
|  |  | Model B: FIGO OR (95% CI) | 1.07 (0.89–1.29) | 1.15 (0.95–1.37) |
|  |  | P value | 0.477 | 0.144 |

^1^ *Camrelizumab-only subgroup (CR=39, PD=7, N=46). Values are median (IQR); P values by Mann–Whitney U test (two-sided).*

^2^ *Spearman rank correlation (two-sided) between biomarker level and FIGO prognostic score in the full cohort (CR=47, PD=13, N=60).*

^3^ *Limited-adjustment logistic regression with outcome PD vs CR in the full cohort (CR=47, PD=13, N=60). Separate models were fitted for HLA-G and OPN. Biomarkers were z-standardized (OR per 1 SD increase). Model A adjusts for immunotherapy agent (Toripalimab vs Camrelizumab). Model B adjusts for FIGO prognostic score (per 1-point increase).*

*Abbreviations: OR, odds ratio; CI, confidence interval; PD, progressive disease; CR, complete response.*

**Supplementary Table S6. Logistic regression model and performance for the plasma-based chemo–immuno score (PD prediction).**

Model: PD (1) vs CR (0). Predictors: pretreatment plasma HLA-G (ng/mL) and OPN (μg/L). Coefficients estimated in the full cohort (n=60). ORs are per 1-unit increase unless otherwise specified; 95% CIs are Wald intervals. AUC CI and Corrected AUC are obtained by bootstrap resampling (1,000 iterations).

**Part A. Model coefficients (original units)**

| **Predictor** | **Beta**  **(log-odds)** | **SE** | **OR** | **95% CI (OR) low** | **95% CI (OR) high** | **P value** |
| --- | --- | --- | --- | --- | --- | --- |
| Intercept | -5.015 | 1.233 | 0.007 | 0.001 | 0.074 | <0.001 |
| HLA-G (ng/mL) | 0.156 | 0.057 | 1.169 | 1.045 | 1.307 | 0.006 |
| OPN  (μg/L) | 0.070 | 0.027 | 1.072 | 1.017 | 1.130 | 0.010 |

**Model formula (original units):**

LP = logit{P(PD)} = -5.015 + 0.156 × HLA-G (ng/mL) + 0.070 × OPN (μg/L)

P(PD) = 1 / [1 + exp(-LP)]

**Part B. Standardized effects (per 1 SD increase)**

| **Predictor** | **Mean** | **SD** | **Beta**  **(log-odds)** | **OR** | **95% CI (OR) low** | **95% CI (OR) high** | **P value** |
| --- | --- | --- | --- | --- | --- | --- | --- |
| HLA-G  (per 1 SD) | 10.909 | 12.534 | 1.957 | 7.075 | 1.740 | 28.768 | 0.006 |
| OPN  (per 1 SD) | 24.962 | 21.300 | 1.484 | 4.410 | 1.433 | 13.568 | 0.010 |

**Model formula (z-score standardized):**

Z_HLA-G_= (HLA-G - 10.909) / 12.534	Z_OPN_= (OPN - 24.962) / 21.300

LP = -1.574+1.957 × Z_HLA-G_ + 1.484 × Z_OPN_

P(PD) = 1 / [1 + exp(-LP)]

**Part C. Discrimination and classification performance**

| **Metric** | **Value** |
| --- | --- |
| AUC (apparent) | 0.908 |
| AUC (bootstrap 95% CI) | 0.813–0.974 |
| Bootstrap optimism-corrected AUC | 0.898 |
| Optimal cutoff (Youden) | 0.25 |
| Sensitivity at optimal cutoff | 0.846 |
| Specificity at optimal cutoff | 0.894 |
| PPV at optimal cutoff | 0.688 |
| NPV at optimal cutoff | 0.955 |
| Accuracy at optimal cutoff | 0.883 |
| TP | 11 |
| FP | 5 |
| TN | 42 |
| FN | 2 |

*Note：Positive and negative predictive values (PPV/NPV) depend on outcome prevalence; values shown were calculated assuming the observed PD prevalence in this cohort (13/60, 21.7%) using Bayes’ theorem: PPV = (Se×Prev)/(Se×Prev + (1−Sp)×(1−Prev)); NPV = (Sp×(1−Prev))/((1−Se)×Prev + Sp×(1−Prev)). To project PPV/NPV to other settings, substitute the expected prevalence (Prev)*

*Abbreviations: AUC, area under the ROC curve; CI, confidence interval; PPV, positive predictive value; NPV, negative predictive value; TP, true positives; FP, false positives; TN, true negatives; FN, false negatives； Se, sensitivity; Sp, specificity; Prev, prevalence.*

**Supplementary Table S7. Clinicopathological and treatment characteristics of patients with evaluable viable tumor tissue^1^ in surgical specimens and available IHC/IF data (CR vs PD)**

| **Characteristic^2^** | **Overall (N=8)** | **CR (n=4)** | **PD (n=4)** | **P value^3^** |
| --- | --- | --- | --- | --- |
| No. of patients | 8 | 4 | 4 |  |
| Age, years | 43.5 (31.5–45.8) | 38.5 (31.5–45.8) | 43.5 (38.2–48.8) | 1.000 |
| FIGO stage |  |  |  | 1.000 |
| III | 3 (38%) | 2 (50%) | 1 (25%) |  |
| IV | 5 (62%) | 2 (50%) | 3 (75%) |  |
| FIGO prognostic score | 13.0 [12.8–13.5] | 12.5 [11.8–13.5] | 13.0 [13.0–13.5] | 0.486 |
| Antecedent pregnancy |  |  |  | — |
| Hydatidiform mole | 2 (25%) | 1 (25%) | 1 (25%) |  |
| Abortion | 4 (50%) | 1 (25%) | 3 (75%) |  |
| Term delivery | 2 (25%) | 2 (50%) | 0 (0%) |  |
| Interval from antecedent pregnancy to chemotherapy, months | 40.0 [6.5–68.5] | 20.5 [4.0–50.5] | 52.0 [34.8–141.0] | 0.343 |
| Pretreatment serum β-hCG, IU/L | 117624  [13344–211632] | 199854  [154345–262347] | 9604  [2016–67962] | 0.200 |
| Largest tumor diameter, cm | 4.0 (3.6–5.5) | 5.3 (3.6–7.0) | 4.0 (3.5–4.4) | 0.686 |
| Pelvic metastasis, n (%) | 1 (12%) | 1 (25%) | 0 (0%) | 1.000 |
| Lung metastasis, n (%) | 8 (100%) | 4 (100%) | 4 (100%) | 1.000 |
| Brain metastasis, n (%) | 5 (62%) | 2 (50%) | 3 (75%) | 1.000 |
| Liver metastasis, n (%) | 0 (0%) | 0 (0%) | 0 (0%) | 1.000 |
| Number of metastatic lesions | 4.0 [2.5–8.2] | 4.0 [2.5–6.0] | 5.5 [2.2–8.2] | 1.000 |
| Prior chemotherapy failure |  |  |  | 0.143 |
| No | 5 (62%) | 4 (100%) | 1 (25%) |  |
| Yes | 3 (38%) | 0 (0%) | 3 (75%) |  |
| Chemotherapy regimen |  |  |  | — |
| EMA/CO | 8 (100%) | 4 (100%) | 4 (100%) |  |
| Anti–PD-1 agent |  |  |  | 0.429 |
| Camrelizumab | 6 (75%) | 4 (100%) | 2 (50%) |  |
| Toripalimab | 2 (25%) | 0 (0%) | 2 (50%) |  |
| Surgery type |  |  |  | — |
| Total hysterectomy + bilateral salpingectomy | 4 (50%) | 2 (50%) | 2 (50%) |  |
| Metastasectomy (brain) | 3 (38%) | 1 (25%) | 2 (50%) |  |
| Metastasectomy (lung) | 1 (12%) | 1 (25%) | 0 (0%) |  |

*^1^ Evaluable viable tumor tissue was defined as a resection specimen with sufficient viable tumor to annotate tumor ROIs and extract quantitative IHC/IF signals. Most other post-treatment surgical specimens showed extensive hemorrhage/necrosis with scant viable tumor and were not suitable for quantitative analyses.*

*^2^ Values are presented as median [interquartile range] or n (%).*

*^3^ P values were calculated using the Mann–Whitney U test for continuous variables and Fisher’s exact test for binary variables. P values were not calculated (—) for multi-category descriptive variables due to the small sample size (N=8). P values were two-sided.*

*Abbreviations: FIGO, International Federation of Gynecology and Obstetrics; hCG, human chorionic gonadotropin; EMA/CO, etoposide + methotrexate + actinomycin D / cyclophosphamide + vincristine; IHC, immunohistochemistry; IF, immunofluorescence; CR, complete response; PD, progressive disease.*

**Supplementary Table S8. Quantitative IHC and IF metrics in surgical specimens with evaluable viable tumor tissue (CR vs PD)**

| **Biomarker / metric** | **Overall (N=8)** | **CR (n=4)** | **PD (n=4)** | **P value** |
| --- | --- | --- | --- | --- |
| Plasma HLA-G, ng/mL | 9.99 [6.86–15.29] | 6.22 [4.60–8.21] | 15.31 [13.87–17.30] | 0.057 |
| HLA-G IHC H-score^1^ | 59.4 [50.8–87.8] | 44.5 [24.5–57.3] | 97.0 [74.2–117.9] | 0.029 |
| HLA-G IF mean fluorescence intensity (MFI)^2^, AU (background-corrected) | 574.12 [418.27–759.26] | 354.75 [215.77–517.09] | 776.47 [687.86–828.35] | 0.040 |
| CD8+ cell density^3^, cells/mm² | 471.30 [403.69–582.14] | 561.98 [482.44–640.89] | 390.05 [276.62–473.00] | 0.200 |
| Plasma osteopontin (OPN), μg/L | 19.67 [15.53–34.25] | 15.29 [12.52–26.34] | 23.77 [20.45–52.49] | 0.320 |
| OPN IHC H-score | 51.6 [40.6–67.3] | 42.3 [40.6–47.6] | 70.7 [54.2–85.7] | 0.486 |
| OPN IF mean fluorescence intensity (MFI), AU (background-corrected) | 302.45 [219.26–474.85] | 188.88 [126.07–259.31] | 484.70 [427.90–556.25] | 0.035 |
| CD163+ cell density, cells/mm² | 254.20 [171.34–425.84] | 129.62 [46.12–219.34] | 432.12 [382.21–446.35] | 0.030 |

*Note: Continuous variables are presented as median [interquartile range] and were compared using the Mann–Whitney U test. P values were two-sided.*

*^1^ IHC H-score ranges from 0 to 300 and was computed as 1×(%1+) + 2×(%2+) + 3×(%3+) within the annotated region of interest.*

*^2^ IF MFI (AU) denotes background-corrected mean fluorescence intensity in arbitrary units.*

*^3^ CD8 and CD163 densities are reported as positive cells per mm².*

**Supplementary Table S9. De-identified patient-level data for cases with evaluable viable tumor tissue in surgical specimens (cases as columns: S01–S08).**

**Part A. Clinical and treatment information**

| **Variable** | **S01** | **S02** | **S03** | **S04** | **S05** | **S06** | **S07** | **S08** |
| --- | --- | --- | --- | --- | --- | --- | --- | --- |
| Response | CR | CR | CR | CR | PD | PD | PD | PD |
| Age, years | 45 | 48 | 30 | 32 | 44 | 24 | 63 | 43 |
| FIGO stage | III | III | IV | IV | IV | IV | IV | III |
| FIGO score | 15 | 13 | 12 | 11 | 13 | 13 | 13 | 15 |
| Antecedent pregnancy | Hydatidiform mole | Abortion | Term delivery | Term delivery | Hydatidiform mole | Abortion | Abortion | Abortion |
| Interval to chemo, months | 94.0 | 36.0 | 1.0 | 5.0 | 44.0 | 7.0 | 384.0 | 60.0 |
| Pretreatment β-hCG, IU/L | 423461 | 191066 | 208642 | 44183 | 17083 | 2125 | 1690 | 220600 |
| Largest tumor, cm | 7.0 | 7.0 | 3.3 | 3.7 | 3.0 | 3.7 | 4.2 | 5.0 |
| Pelvic mets | Yes | No | No | No | No | No | No | No |
| Lung mets | Yes | Yes | Yes | Yes | Yes | Yes | Yes | Yes |
| Brain mets | No | No | Yes | Yes | Yes | Yes | Yes | No |
| Liver mets | No | No | No | No | No | No | No | No |
| No. metastatic lesions | 9 | 1 | 3 | 5 | 3 | 8 | 9 | 1 |
| Prior chemo failure | No | No | No | No | No | Yes | Yes | Yes |
| Chemo regimen | EMA/CO | EMA/CO | EMA/CO | EMA/CO | EMA/CO | EMA/CO | EMA/CO | EMA/CO |
| Anti–PD-1 agent | Camrelizumab | Camrelizumab | Camrelizumab | Camrelizumab | Camrelizumab | Camrelizumab | Toripalimab | Toripalimab |
| Surgery type | Total hysterectomy + bilateral salpingectomy | Total hysterectomy + bilateral salpingectomy | Metastasectomy (lung) | Metastasectomy (brain) | Metastasectomy (brain) | Total hysterectomy + bilateral salpingectomy | Metastasectomy (brain) | Total hysterectomy + bilateral salpingectomy |

**Part B. Quantitative biomarkers (IHC/IF and plasma)**

| **Variable** | **S01** | **S02** | **S03** | **S04** | **S05** | **S06** | **S07** | **S08** |
| --- | --- | --- | --- | --- | --- | --- | --- | --- |
| Plasma HLA-G, ng/mL | 3.63 | 10.31 | 7.51 | 4.92 | 23.18 | 15.27 | 9.67 | 15.35 |
| HLA-G IHC H-score^1^ | 2.2 | 57.1 | 31.9 | 57.8 | 115.5 | 125.0 | 78.6 | 61.0 |
| HLA-G IF MFI^2^, AU | 180.0 | 481.8 | 227.7 | 622.95 | 880.7 | 810.9 | 742.05 | 525.3 |
| CD8 density^3^, cells/mm² | 696.2 | 501.5 | 622.45 | 425.25 | 568.7 | 89.5 | 339.0 | 441.1 |
| Plasma OPN, μg/L | 15.78 | 5.69 | 14.8 | 58.03 | 26.32 | 131.01 | 18.11 | 21.22 |
| OPN IHC H-score | 42.5 | 42.1 | 36.0 | 62.9 | 34.9 | 100.6 | 60.7 | 80.7 |
| OPN IF MFI, AU | 288.3 | 128.1 | 120.0 | 249.65 | 465.0 | 711.8 | 316.6 | 504.4 |
| CD163 density, cells/mm² | 238.2 | 46.2 | 45.9 | 213.05 | 444.7 | 270.2 | 451.3 | 419.55 |

*Notes: Case IDs (S01–S08) are study-specific and do not correspond to clinical identifiers.*

*^1^IHC H-score ranges from 0 to 300 (1×%1+ + 2×%2+ + 3×%3+) within the annotated region of interest.*

*^2^IF MFI denotes background-corrected mean fluorescence intensity in arbitrary units (AU).*

*^3^Cell densities are reported as positive cells per mm².*

*Abbreviations: IHC, immunohistochemistry; IF, immunofluorescence; MFI, mean fluorescence intensity; hCG, human chorionic gonadotropin; mets, metastasis; CR, complete response; PD, progressive disease.*

**Supplementary Table S10 Detailed treatment timeline of the study cohort (shown as EXCEL)**

**Supplementary Table S11. Descriptive comparison of clinical characteristics between the exploratory tissue subset and the Overall cohort**

| **Characteristic** | **Overall cohort (N=60)** | **Mechanistic validation tissue subset (n=8)** | **P value*** |
| --- | --- | --- | --- |
| **Disease status** |  |  | 1.000 |
| Primary | 46 (76.7) | 6 (75.0) |  |
| Recurrent | 14 (23.3) | 2 (25.0) |  |
| Age, years | 33.0 [29.0–38.0] | 43.5 [31.5–45.8] | 0.056 |
| **FIGO stage** |  |  | 0.671 |
| I | 7 (11.6) | 0 (0.0) |  |
| II | 3 (5.0) | 0 (0.0) |  |
| III | 25 (41.7) | 3 (37.5) |  |
| IV | 25 (41.7) | 5 (62.5) |  |
| FIGO prognostic score | 13.0 [10.0–15.0] | 13.0 [12.8–13.5] | 0.446 |
| **Antecedent pregnancy** |  |  | 0.355 |
| Hydatidiform mole | 19 (31.7) | 2 (25.0) |  |
| Abortion/miscarriage | 18 (30.0) | 4 (50.0) |  |
| Term delivery | 23 (38.3) | 2 (25.0) |  |
| Interval from antecedent pregnancy to treatment, months | 31 [12–55] | 40 [6–68] | 0.854 |
| Pretreatment serum β-hCG, IU/L | 53,333 [2,981–261,830] | 117,624 [13,344–211,632] | 0.795 |
| Maximum tumor diameter, cm | 5.0 [2.8–7.0] | 4.0 [3.6–5.5] | 0.854 |
| Pelvic metastasis, yes | 8 (14.0) | 1 (12.5) | 1.000 |
| Lung metastasis, yes | 46 (80.7) | 8 (100.0) | 0.332 |
| Other metastatic sites, yes | 10 (17.5) | 2 (25.0) | 0.619 |
| Number of metastatic lesions | 1.0 [1.0–5.0] | 4.0 [2.5–8.2] | 0.094 |
| History of chemotherapy failure, yes | 25 (43.9) | 3 (37.5) | 1.000 |
| **Immunotherapy agent** |  |  | 1.000 |
| Camrelizumab | 46 (76.7) | 6 (75.0) |  |
| Toripalimab | 14 (23.3) | 2 (25.0) |  |

*Data are presented as median [interquartile range] for continuous variables and n (%) for categorical variables.*

**P values compare the mechanistic validation tissue subset with the remaining cohort using Mann–Whitney U test for continuous variables and Fisher’s exact test or Fisher–Freeman–Halton exact test for categorical variables.*

*Abbreviations: FIGO, International Federation of Gynecology and Obstetrics; hCG, human chorionic gonadotropin; IU/L, international units per liter; CR, complete response; PD, progressive disease;*

**Supplementary Table S12. Sensitivity analysis of differential protein selection thresholds**

Analysis based on the plasma proteomics expression matrix from the nested discovery subset (21 CR and 9 PD samples). Differential statistics were recalculated from log2 intensities; P values were adjusted using the Benjamini-Hochberg method.

**A. Threshold-level sensitivity analysis**

| **Threshold ID** | **Differential selection criterion** | **No. of proteins retained** | **Upregulated in PD** | **Downregulated in PD** | **HLA-G retained** | **HLA-G log2FC** | **HLA-G FDR** | **SPP1/OPN retained** | **SPP1 log2FC** | **SPP1 FDR** |
| --- | --- | --- | --- | --- | --- | --- | --- | --- | --- | --- |
| T1 | FDR < 0.05 only | 674 | 155 | 519 | Yes | 3.945 | 2.29E-07 | Yes | 3.043 | 3.54E-06 |
| T2 | \|log2FC\| ≥ 0.5 and FDR < 0.05 | 673 | 154 | 519 | Yes | 3.945 | 2.29E-07 | Yes | 3.043 | 3.54E-06 |
| T3 | \|log2FC\| ≥ 1.0 and FDR < 0.05 | 512 | 98 | 460 | Yes | 3.945 | 2.29E-07 | Yes | 3.043 | 3.54E-06 |
| T4 | \|log2FC\| ≥ 1.5 and FDR < 0.05 | 374 | 61 | 313 | Yes | 3.945 | 2.29E-07 | Yes | 3.043 | 3.54E-06 |
| T5 | \|log2FC\| ≥ 1.0 and FDR < 0.01 | 400 | 78 | 322 | Yes | 3.945 | 2.29E-07 | Yes | 3.043 | 3.54E-06 |
| T6 | \|log2FC\| ≥ 2.0 and FDR < 0.01 | 290 | 44 | 246 | Yes | 3.945 | 2.29E-07 | Yes | 3.043 | 3.54E-06 |

**B. Candidate-level robustness**

| **Protein** | **Mean CR** | **Mean PD** | **log2FC PD vs CR** | **P value** | **FDR** | **Direction** | **T1** | **T2** | **T3** | **T4** | **T5** | **T6** |
| --- | --- | --- | --- | --- | --- | --- | --- | --- | --- | --- | --- | --- |
| HLA-G | 20.503 | 24.449 | 3.945 | 2.11E-09 | 2.29E-07 | Up in PD | Yes | Yes | Yes | Yes | Yes | Yes |
| SPP1 | 22.277 | 25.320 | 3.043 | 7.58E-08 | 3.54E-06 | Up in PD | Yes | Yes | Yes | Yes | Yes | Yes |

*Note: HLA-G and SPP1/OPN remained significantly upregulated in PD patients and were retained under all alternative differential selection thresholds tested. T1, FDR < 0.05 only; T2, |log2FC| >= 0.5 and FDR < 0.05; T3, |log2FC| >= 1.0 and FDR < 0.05; T4, |log2FC| >= 1.5 and FDR < 0.05; T5, |log2FC| >= 1.0 and FDR < 0.01; T6, |log2FC| >= 2.0 and FDR < 0.01.*

*Abbreviations: CR, complete response; PD, progressive disease; FDR, false discovery rate; log2FC, log2 fold change; OPN, osteopontin; SPP1, secreted phosphoprotein 1.*

**Supplementary Table S13. Compartment-specific immunofluorescence quantification of CD8+ T cells and CD163+ macrophages**

| **Variable** | **Compartment** | **S01** | **S02** | **S03** | **S04** | **S05** | **S06** | **S07** | **S08** |
| --- | --- | --- | --- | --- | --- | --- | --- | --- | --- |
|  | **Response** | **CR** | **CR** | **CR** | **CR** | **PD** | **PD** | **PD** | **PD** |
| CD8+ T-cell density, cells/mm² | Intratumoral | 696.2 | 501.5 | 622.45 | 425.25 | 568.7 | 89.5 | 339.0 | 441.1 |
| CD8+ T-cell density, cells/mm² | Peritumoral | 737.2 | 469.3 | 601.5 | 561.8 | 606.1 | 57.4 | 187.9 | 265.6 |
| CD163+ macrophage density, cells/mm² | Intratumoral | 238.2 | 46.2 | 45.9 | 213.05 | 444.7 | 270.2 | 451.3 | 419.55 |
| CD163+ macrophage density, cells/mm² | Peritumoral | 136.8 | 34.7 | 53.7 | 167.6 | 536.5 | 234.5 | 477.5 | 449.6 |

*Notes: Case IDs (S01-S08) are study-specific and do not correspond to clinical identifiers. S01-S04 are complete response (CR) cases and S05-S08 are progressive disease (PD) cases.*

*Intratumoral compartment was defined as viable tumor-cell regions within the annotated tumor boundary. Peritumoral compartment was defined as the stromal band extending 200 µm outward from the tumor invasive margin, excluding necrosis, hemorrhage, tissue folds, edge artifacts, and non-tumor/non-stromal regions.*

*Cell densities are reported as positive cells per mm². Abbreviations: IF, immunofluorescence; CR, complete response; PD, progressive disease.*
