# Supplementary material for: Integrated plasma proteomics identifies HLA-G and osteopontin as circulating biomarkers of resistance to PD-1 inhibitor plus chemotherapy in choriocarcinoma
Source: Front Immunol. 2026 Aug 4;17:1817862. doi: 10.3389/fimmu.2026.1817862 (PMC13481723; doi:10.3389/fimmu.2026.1817862)
Supplement: Supplementary file 3 [file Table3.docx]

**Supplementary Fig. 1 | Network-based prioritization and regulatory architecture of the resistance-associated signature**

(A) Protein–protein interaction (PPI) network constructed for upregulated resistance-associated proteins using the Search Tool for the Retrieval of Interacting Genes/Proteins (STRING) and visualized in Cytoscape. Interactions were retrieved using a minimum required interaction score of 0.4 with all evidence channels enabled (including experimental, curated database and text-mining evidence). Node color represents degree (number of connections). Immune response–related genes are indicated as triangles and other upregulated genes as rounded rectangles. A densely connected module was identified using Molecular Complex Detection (MCODE) in Cytoscape (degree cutoff = 2; node score cutoff = 0.2; K-core = 2; max depth = 100; haircut enabled), and the extracted module is shown on the right.

(B) Spearman correlation network of immune response–related differentially expressed proteins, comprising 12 proteins upregulated and 5 proteins downregulated in progressive disease, with edges shown for |ρ| ≥ 0.2. Edge color denotes correlation direction (red, positive; blue, negative) and edge width indicates the absolute correlation magnitude. Node size represents average expression, and nodes are annotated by functional categories (acute-phase inflammation, antigen presentation, cytokine/chemokine signaling, and immune checkpoint/adherence).

(C) Integrated transcription factor–messenger RNA–microRNA (TF–mRNA–miRNA) regulatory network for the upregulated immune response-related signature. Predicted miRNA–mRNA interactions were obtained from miRWalk, and transcription factor–mRNA regulatory relationships were curated from TRRUST. Nodes denote transcription factors (blue diamonds), messenger RNAs (red ellipses) and microRNAs (green inverted triangles); edges indicate regulatory interactions.

*Abbreviations: MCODE, Molecular Complex Detection; miRNA, microRNA; mRNA, messenger RNA; PPI, protein–protein interaction; TF, transcription factor.*

**Supplementary Fig. 2 | Feature selection by least absolute shrinkage and selection operator (LASSO) regression.**

a, Three-fold cross-validation curve for the LASSO logistic regression model showing the cross-validated deviance across values of log(λ). Dots indicate the mean cross-validated deviance and error bars indicate ±1 s.e. The vertical dashed lines denote λ_min (minimum mean cross-validated deviance) and λ_1se (largest λ within 1 s.e. of the minimum). Numbers along the top indicate the number of non-zero coefficients at each λ.

b, LASSO coefficient profiles for candidate predictors plotted against log(λ). Each curve represents the coefficient trajectory of a feature as regularization increases; the vertical dashed lines indicate λ_min and λ_1se, respectively.

*Abbreviations: LASSO, least absolute shrinkage and selection operator; CV, cross-validation; s.e., standard error.*
